# Artificial Neural Network Modeling of Nanostructured Lipid Carriers Containing 5-O-Caffeoylquinic Acid-Rich *Cratoxylum formosum* Leaf Extract for Skin Application

**DOI:** 10.34172/apb.2022.082

**Published:** 2021-10-06

**Authors:** Abhiruj Navabhatra, Adelheid Brantner, Bancha Yingngam

**Affiliations:** ^1^Department of Pharmacology, College of Pharmacy, Rangsit University, Pathum Thani, 12000, Thailand.; ^2^Department of Pharmacognosy, Institute of Pharmaceutical Sciences, University of Graz, Universitaetsplatz 4/1, A-8010, Graz, Austria.; ^3^Department of Pharmaceutical Chemistry and Technology, Faculty of Pharmaceutical Sciences, Ubon Ratchathani University, Ubon Ratchathani, 34190, Thailand.

**Keywords:** Artificial neural network, Chemical analysis, Computer modeling, Nanostructured lipid carriers, Safety testing

## Abstract

***Purpose:*** To investigate the in vitro anti-skin-aging properties of *Cratoxylum formosum* extract and encapsulate this plant extract in nanostructured lipid carriers (CFE-NLCs) for dermal application.

***Methods:*** The biological properties of the plant extract, including enhanced procollagen type I synthesis and anti-matrix metalloproteinase activity, were evaluated to assess its cosmetic benefits. An artificial neural network (ANN) coupled with K-fold cross-validation was applied to investigate the effects of the formulants and optimize the CFE-NLCs. The physicochemical properties, percutaneous absorption, and irritation potential of the CFE-NLCs were analyzed.

***Results:*** Liquid chromatography-mass spectrometry analysis revealed that CFE contained 5-O-caffeoylquinic acid as the vital constituent. Appropriate skin-care properties were also demonstrated with respect to enhanced type I procollagen synthesis and the inhibition of MMP-1, MMP-3, and MMP-9 in primary human dermal fibroblasts. The optimal CFE-NLCs exhibited better skin absorption and biocompatibility and lower irritation potential than the free botanical extract solution.

***Conclusion:*** The findings obtained highlight CFE-NLCs as promising skin-care ingredients.

## Introduction

Recent trends in natural product research have greatly improved the prevention of skin aging and other cellular oxidative stresses.^1^ However, low stability, a lack of skin absorption, and irritation frequently impede the use of plant extracts containing bioactive molecules in cosmetics. The most popular approach to overcome these limitations is the engineered nanoscale formulation strategy, which has introduced new challenges in the cosmetic field.^2^

The young leaves of *Cratoxylum formosum* (Jacq.) Benth. & Hook.f. ex Dyer (Hypericaceae family) are rich in phenolic compounds, among which 5-*O*-caffeoylquinic acid (5CQA) has been identified as the major component.^3,4^ Studies have reported that *C. formosum* extract (CFE) exhibits antioxidant activity,^3^ cellular protection against oxidative stress,^4^ antibacterial activity and anti-nonmelanoma skin cancer activity when used in aqueous form to synthesize ZnO nanosheets,^5^ antigenotoxicity,^6^ and anti-inflammatory properties.^7^

Interestingly, 5CQA, the main component of *C. formosum* leaf extract, exhibits vital biological activities, such as the promotion of type 17 collagen production,^8^ the prevention of anionic surfactant-induced itching in the epidermis,^9^ protection against ultraviolet-A-induced oxidative stress,^10^ and antioxidant, antibacterial, anti-inflammatory, antimutagenic, anticancer, and immunomodulatory properties.^11^ Thus, evidence of the health benefits of 5CQA and 5CQA-rich plant extracts supports the possible application of CFE to dermocosmetics.

Despite its cosmetic potential, the use of bioactive molecules of CFE is most often impaired by stability constraints or insufficient dermal absorption. Specifically, 5CQA readily undergoes oxidation, is unstable at high temperatures (100–200°C) and neutral to alkaline environments (pH ≥ 7), and undergoes isomerization and methylation during processing and storage.^12,13^ Xue et al^14^ reported that 5CQA was unstable: *cis*-5CQA (24% relative content) and methylated 5CQA (1% relative content) degraded after seven days of storage at room temperature under light irradiation. In addition, 5CQA is hydrolyzed to quinic acid, caffeic acid, and *cis*-caffeic acid when processed in boiling phosphate buffer at pH ≥ 7.^13^ Conversely, 5CQA remains stable at pH ≤ 6, even when the compound is heated for more than 6 h in phosphate buffer.^13^ In addition, the skin can absorb only a limited amount of 5CQA.^15^ The low bioavailability is attributed to the hydrophilicity of 5CQA, which hinders it from passing the lipophilic membrane barrier.^16^ In addition, 5CQA has a short half-life and high distribution and elimination rates in the human body.^10^ These properties restrict the potential use of this plant extract, especially in water-based cosmetic products.

Studies have shown that the chemical stability of sensitive molecules can be improved by nanoscale particles, such as transfersomes, nanoparticles, and nanostructured lipid carriers (NLCs).^17-20^ In the cosmetic and pharmaceutical industries, NLCs have received much attention for use in conserving biological properties, controlling release, and enhancing bioavailability.^21^ In our preliminary study,^22^ some NLC factors were screened to select their relevant ranges for encapsulation of phenolics from CFE. Unfortunately, the implementation of response surface methodology (RSM) neither successfully achieved the required formulation nor modeled the complicated relationship between the studied variables. This result is attributed to the limits of RSM in overcoming the simple relationship between the formulation parameters in a linear or quadratic relationship. Therefore, the optimum NLCs containing plant extracts rich in 5CQA for dermal targeting have yet to be identified, and most current studies still apply a one-factor-at-a-time approach in formulation optimization.^15^

 Thus, an artificial neural network (ANN), an intelligent machine learning tool, is chosen instead of RSM to solve complex and nonlinear relationships related to formulation processes without a prior model option. This versatile technology has been used by scientists from various disciplines and has emerged as a particularly attractive technology for the cosmetic industry. This study aimed to (i) investigate the in vitro anti-skin-aging properties of 5CQA-rich CFE, (ii) optimize the encapsulation of this plant extract in nanostructured lipid carriers (CFE-NLCs), and (iii) evaluate its cytotoxicity and irritation effects for dermal applications.

## Materials and Methods

###  Chemicals

The following chemicals were used in this study: glyceryl behenate (Gattefossé, Saint-Priest, France); poloxamer 188, polysorbate 80, ˪-ascorbic acid, and 5CQA (Sigma-Aldrich Company, St. Louis, MO, USA); glyceryl monostearate (BDH Chemicals Ltd, Poole, England); caprylic/capric triglycerides (INOLEX Company, Bangkok, Thailand); H_2_O_2_ (Carlo Erba Reagents S.A.S., Val de Reuil, France); and Dulbecco’s modified Eagle’s medium (DMEM), fetal bovine serum (FBS), penicillin-streptomycin, 0.25% trypsin-EDTA, and 3-(4,5-dimethylthiazol-2-yl)-2,5-diphenyltetrazolium bromide (MTT) (Gibco-BRL, Grand Island, NY, USA). All the chemicals used were of analytical grade.

###  Preparation of plant extract

Cultivated young leaves of *C. formosum* were collected from a local field in Ubon Ratchathani province, Thailand. The plant was identified by comparison to the voucher specimen BCY No. 022 deposited in the herbarium of the Faculty of Pharmaceutical Sciences, Ubon Ratchathani University. The leaves were dried at 50°C and sifted through a 40-mesh screen sieve. Extraction of the plant powder was performed using a heat-reflux method (38.83% ethanol in water, 59.76°C, 5.50 h) according to the optimized extraction conditions previously reported by the authors.^3^ Ethanol was removed under vacuum, and the resulting CFE in aqueous solution was dried in a freeze dryer (Martin Christ GmbH, Osterode, Germany) before use.

###  Identification and quantification of phytochemicals

####  High-performance liquid chromatography-photodiode array detection-electrospray ionization-tandem mass spectrometry (HPLC-PDA-ESI-MS)

Polyphenols in CFE were identified using HPLC-PDA-ESI-MS analysis.^4^ The instrument consisted of a Dionex UltiMate 3000 RS LC system connected to an LTQ XL linear ion-trap mass spectrometer, and data processing was performed by Xcalibur 2.2 software (Thermo Fisher Scientific Inc., Waltham, MA, USA). The sample (5 mg/mL) (5 µL) was injected into a LiChrospher^®^ RP-18 column (150 × 4.6 mm, inner diameter 5 µm) using 0.1% (*v/v*) formic acid in water (A) and acetonitrile (B) as the eluent. For chromatographic separation, the eluent proportion began at 5% B (5 minutes), increased from 5% B to 50% B (45 minutes), remained constant at 50% B (10 min), and returned to 5% B (2 minutes). The eluent flow was 0.5 mL/min, and the column temperature was maintained at 35°C. The PDA range was set at 190 to 500 nm. Specific conditions for mass spectrometric analysis were set as follows: electrospray ionization, negative and positive ion modes; capillary temperature, 330°C; source heater temperature, 250°C; sheath gas flow, 50 arbitrary units; auxiliary gas flow, 10 arbitrary units; capillary voltage, –45 V; source voltage of negative ion mode, 3 kV; source voltage of positive ion mode, 3.5 kV; source current, 100 µA; normalization collision energy, 35%; mass range, 50–2000 atomic mass units. MS^n^ spectra were generated by collision-induced dissociation using helium as the collision gas. The polyphenolic components of CFE were tentatively identified by comparing their retention times, ultraviolet spectra, and MS^n^ fragmentation patterns to those of authentic compounds and those reported in the literature.

####  HPLC-PDA analysis

The 5CQA content of CFE was determined using HPLC (Dionex Ultimate 3000 UHPLC, Thermo Fisher Scientific Company, USA).^3^ The sample (10 µL) was injected into a Hypersil Gold C_18_ column (250 × 4.6 mm, inner diameter 5 µm). The mobile phase consisted of 50 mM phosphoric acid in water (solvent A) and acetonitrile (solvent B) at a flow rate of 0.7 mL/min. The stepwise elution was performed as follows: 5% B for 5 min, 5% B to 50% B in 45 min, 50% B for 10 minutes, and 50% B to 5% B in 2 minutes. The column temperature was 35 ± 0.1°C, and detection was performed at 320 nm. 5CQA was quantified by comparing the peak area obtained with the calibration curve of the authentic chemical.

###  Biological properties of CFE

####  Cytotoxicity of CFE in primary human dermal fibroblasts (HDFn)

HDFn cells were grown in DMEM supplemented with 10% FBS, 1% GlutaMax, and 1% penicillin-streptomycin solution according to the method previously reported by this group of authors.^23^ HDFn cells were seeded overnight at a density of 5 × 10^3^ cells/well on a 96-well culture plate and then treated with various concentrations of CFE (0–5000 µg/mL) for 24 hours. The culture medium was replaced with 0.5 mg/mL MTT solution, and the cells were incubated for 3 hours at 37°C. The MTT solution was removed, and 100 μL of dimethyl sulfoxide (DMSO) was added to solubilize the formazan product. The intensity of the formazan product was measured at 570 nm using a PowerWave XS2 microplate reader (BioTek, Winooski, VT, USA). The relative cell viability of the treatment group was calculated as a percentage of that of the vehicle control group.

####  Procollagen type I assay

HDFn cells were incubated in 6‐well plates at a density of 3 × 10^5^cells/well overnight. The cells were treated with varying concentrations of CFE (0–100 µg/mL) in serum-free medium for 48 hours. After incubation, the cell-free supernatants were collected from each well, and collagen levels were determined using a Human Procollagen I α1 ELISA Kit (Abcam, Cambridge, MA, USA) following the instructions of the manufacturer. ˪-Ascorbic acid (200 μg/mL) was used as a positive control, and the results were normalized to confluent cell numbers.

####  Determination of anti-MMP secretion

HDFn cells were seeded into 6‐well plates at a density of 3 × 10^5^cells/well and cultivated for 24 hours. The cells were treated with different concentrations of CFE (0–100 µg/mL) or with 200 μg/mL ˪-ascorbic acid in serum-free medium for 1 h before the addition of lipopolysaccharides (LPSs) (1 µg/mL). After 12 and 24 hours of LPS exposure, the cell-free supernatants were collected from each well and centrifuged at 15 000 rpm and 4°C for 15 min. The MMP-1, MMP-3, and MMP-9 levels in the culture media were measured using MMP-1, MMP-3, and MMP-9 ELISA kits (Sigma-Aldrich, St. Louis, MO, USA) according to the manufacturer’s instructions.

###  Preparation of CFE-NLCs

The CFE-NLCs were prepared by a high-shear ultrasonic homogenization method.^20^ The NLCs comprised solid lipids (glyceryl behenate:glyceryl monostearate = 1:1), liquid lipids (caprylic/capric triglyceride), emulsifiers (polysorbate 80:poloxamer 188 = 1:1), and CFE as an active ingredient. The lipid and aqueous phases were separately heated at 80°C for 10 minutes. The preparation temperature did not decompose 5CQA. The hot aqueous phase containing CFE and emulsifier was then added to the lipid phase followed by 10 min of homogenization at 6000 rpm (Ultra Turrax T25, IKA-Werke, Germany). The particle size in the obtained coarse emulsion was then reduced by applying an ultrasonic cell disruption device (Vibra-Cell Ultrasonic Liquid Processors, Sonics & Materials, Inc., 20 kHz, Newtown, CT, USA) for 10 min (active for 2 seconds at intervals of 2 s, 50 W). The resulting nanoparticles were cooled to room temperature, stored in N_2_-flushed glass bottles, and shielded from light for further study. The above procedure was also used to prepare blank NLCs without CFE.

###  Experimental design

A central composite rotatable design (CCRD) was applied to optimize the formulation parameters of CFE-NLCs. This statistical design consisted of a 16-factorial design, 8 axial points, and 6-center-point replication. Factorial and axial points were used to estimate the curvature of the model, whereas replicate experiments were used to assess the pure error sum of squares. The independent variables included the proportions of solid lipids (*X*_1_), liquid lipids (*X*_2_), emulsifiers (*X*_3_), and CFE (*X*_4_) in the mixture. The hydrodynamic diameter (*Y*_1_), polydispersity index (PDI) (*Y*_2_), zeta potential (*Y*_3_), and entrapment efficiency (%EE) of 5CQA in NLCs (*Y*_4_) were assigned as the dependent variables because they are essential features of the nanoparticles. The coded independent variables in the CCRD were calculated from Eq. (1). Table 1 summarizes the layout of the design matrix.

**Table 1:** Independent variables, symbols, coded levels and actual values of the experimental design

**Independent variable**	**Symbol**	**Levels**				
		**–2**	**–1**	**0**	**+1**	**+2**
Solid lipid content (glyceryl behenate+glyceryl monostearate mixture (1:1)) (wt%)	*X*_1_	0.25	3.50	6.75	10.00	13.25
Liquid lipid content (caprylic/capric triglyceride) (% of total lipid content)	*X*_2_	0.00	15.00	30.00	45.00	60.00
Emulsifier (polysorbate 80+poloxamer 188 (1:1)) (wt%)	*X*_3_	0.50	2.00	3.50	5.00	6.50
CFE (wt%)	*X*_4_	0.13	0.75	1.38	2.00	2.63
Dependent variables				Constraints		
Particle size (nm)	*Y*_1_			Minimize		
PDI	*Y*_2_			Minimize		
Zeta potential value (mV)	*Y*_3_			Minimize		
%*EE* of 5CQA (%)	*Y*_4_			Maximize		


(1) $$x_{i}=\left(\frac{X_{i}-X_{0}}{\Delta X_{i}}\right)$$

where *x*_i_, *X*_i_, and *X*_o_ are the coded value, corresponding actual value, and actual value in the center of the domain, respectively. *∆X* is the increment of *X*_i_, corresponding to a change of 1 unit in *x*. The range of each independent variable was selected based on the results obtained from preliminary experiments.^22^ A total of 30 experimental runs were conducted, and the conditions are summarized in Table 2. All experiments were randomized to mitigate the effects of unintended external variables.

**Table 2:** Layout of the CCRD and the corresponding response values for the preparation of CFE-NLCs

**No.**	**Independent variable**				**Dependent variable**			
	***X***_1_ ** (wt%)**	***X***_2_ ** (% total lipid content)**	***X***_3_ ** (wt%)**	***X***_4_ ** (wt%)**	***Y***_1_ ** (nm)**	***Y***_2_	***Y***_3_ ** (mV)**	***Y***_4_ ** (%)**
1	6.75	30.00	6.50	1.38	59.25 ± 0.73^a,h^	0.17 ± 0.02^a^	–11.93 ± 2.24^a^	75.83 ± 5.54^a,c,e,f^
2*	6.75	30.00	3.50	1.38	99.98 ± 1.64^b^	0.17 ± 0.01^b,d^	–10.48 ± 2.64^a^	78.31 ± 1.17^a,c,e,f^
3	6.75	30.00	3.50	0.13	99.29 ± 1.27^b^	0.18 ± 0.03^a^	–10.05 ± 0.60^a^	69.10 ± 4.13^a,c,e,f^
4	3.50	15.00	2.00	0.75	111.13 ± 2.53^b,c^	0.22 ± 0.02^a^	–11.16 ± 1.23^a^	56.80 ± 7.85^a,d,e^
5	6.75	30.00	3.50	2.63	104.00 ± 1.63^b,c^	0.19 ± 0.02^a^	–12.70 ± 0.93^a^	58.44 ± 1.78^a,d,e^
6	0.25	30.00	3.50	1.38	76.48 ± 4.37^a^	0.41 ± 0.27^a^	–14.89 ± 3.40^a^	67.80 ± 0.14^a,c,e,f^
7	6.75	30.00	0.50	1.38	489.33 ± 5.92^d^	0.45 ± 0.21^a,d^	–14.79 ± 1.49^a^	65.64 ± 3.94^a,c,e^
8	10.00	15.00	5.00	2.00	151.43 ± 2.37^e^	0.27 ± 0.01^a^	–6.68 ± 4.49^b,c^	40.30 ± 1.88^b,d,e^
9	13.25	30.00	3.50	1.38	240.00 ± 7.39^f^	0.26 ± 0.06^a^	–9.85 ± 0.35^a^	57.63 ± 0.41^a,c,d,e^
10*	6.75	30.00	3.50	1.38	111.20 ± 5.88^b,c^	0.18 ± 0.03^a^	–10.89 ± 3.67^a^	77.91 ± 4.18^a,c,e,f^
11*	6.75	30.00	3.50	1.38	106.11 ± 7.14^b,c^	0.18 ± 0.02^a^	–10.72 ± 1.45^a^	76.38 ± 1.79^a,c,e,f^
12*	6.75	30.00	3.50	1.38	95.76 ± 5.29^b^	0.18 ± 0.02^a^	–10.07 ± 2.65^a^	77.31 ± 1.87^a,c,e,f^
13	10.00	45.00	5.00	0.75	120.57 ± 2.07^c^	0.21 ± 0.02^a^	–6.16 ± 1.65^b^	64.04 ± 2.21^a,d,e^
14	3.50	15.00	2.00	2.00	120.50 ± 1.85^c^	0.30 ± 0.06^a^	–12.95 ± 1.84^a,c^	51.82 ± 3.99^b,d^
15	10.00	45.00	2.00	2.00	243.00 ± 4.28^f^	0.19 ± 0.05^a^	–12.73 ± 1.21^a^	62.22 ± 1.65^a,d,e^
16	10.00	45.00	5.00	2.00	117.00 ± 2.14^c,g^	0.03 ± 0.00^c^	–10.70 ± 3.22^a^	65.61 ± 5.39^a,c,e^
17	10.00	15.00	5.00	0.75	121.39 ± 1.74^c^	0.23 ± 0.01^a^	–5.76 ± 1.79^b,c^	45.13 ± 3.01^b,d^
18	3.50	45.00	5.00	0.75	53.25 ± 1.13^h^	0.23 ± 0.03^a^	–8.58 ± 2.68^a^	73.28 ± 1.97^a,c,e,f^
19	10.00	15.00	2.00	0.75	459.25 ± 19.70^i^	0.33 ± 0.13^a^	–12.71 ± 2.69^a^	66.91 ± 7.10^a,c,e^
20*	6.75	30.00	3.50	1.38	99.76 ± 1.78^b^	0.17 ± 0.03^a,b^	–10.69 ± 1.45^a^	75.78 ± 5.18^a,c,e,f^
21	10.00	15.00	2.00	2.00	456 ± 12.88^i^	0.34 ± 0.09^a^	–12.01 ± 1.86^a^	59.71 ± 7.72^a,d,e^
22	3.50	15.00	5.00	2.00	76.48 ± 1.26^a^	0.42 ± 0.02^a^	–7.99 ± 2.00^a^	77.66 ± 1.20^a,c,e,f^
23	6.75	0.00	3.50	1.38	147.00 ± 2.27^e^	0.26 ± 0.01^a^	–10.85 ± 0.53^a^	66.06 ± 4.79^a,c,e^
24	3.50	45.00	2.00	2.00	82.21 ± 1.05^a,b^	0.18 ± 0.02^a^	–15.06 ± 1.67^a^	77.70 ± 4.50^a,c,e,f^
25	10.00	45.00	2.00	0.75	235.29 ± 4.35^f^	0.19 ± 0.04^a^	–8.02 ± 3.93^a^	59.61 ± 1.38^a,d,e^
26	3.50	15.00	5.00	0.75	88.85 ± 1.28^a,b^	0.36 ± 0.03^a^	–7.71 ± 0.92^a^	63.80 ± 5.41^a,c,d,e^
27	3.50	45.00	2.00	0.75	90.94 ± 1.56^a,b^	0.20 ± 0.02^a^	–12.94 ± 0.69^a^	74.39 ± 5.91^a,c,e,f^
28	3.50	45.00	5.00	2.00	57.68 ± 1.39^a,h^	0.27 ± 0.04^a^	–10.62 ± 1.20^a^	77.62 ± 5.40^a,c,f^
29*	6.75	30.00	3.50	1.38	96.78 ± 3.05^b^	0.16 ± 0.09^b,d^	–10.87 ± 1.84^a^	80.76 ± 3.12^a,c,f^
30	6.75	60.00	3.50	1.38	124.13 ± 3.52^c^	0.19 ± 0.02^a^	–13.58 ± 1.33^a^	66.90 ± 1.60^a,c,e^

Data are expressed as the mean ± SD (*n* = 3). Values sharing the same letter in each column do not exhibit statistically significant differences (*P* > 0.05). * Center point.

###  ANN modeling

An ANN with K-fold cross-validation was applied to the above 30 experimental datasets using JMP Pro 14 software (SAS Institute Inc., NC, USA).^24^ This approach randomly divided the initial dataset into equal portions, called folds. One fold was used for testing, and the remaining k−1 folds were used for training. After repeating the algorithm on all the folds, the model was fit on the training dataset and estimated on the testing dataset. The best model was chosen by achieving the minimum error on the basis of different error-estimating statistical tools. The ANN architecture was defined as A-B-C, representing the number of neurons in the input, hidden, and output layers, respectively. A hyperbolic tangent (*TanH*) function (Eq. 2) was applied between the input and hidden layers, whereas a linear transfer function was used between the hidden layer and the output layer.


(2) $$\int x=\mathrm{Tan}H\left(x\right)=\frac{\left(e^{x}-e^{-x}\right)}{\left(e^{x}+e^{-x}\right)}$$

The optimum ANN model was calculated by changing the number of neurons in the hidden layer and choosing the minimum root-mean-square error (RMSE), mean absolute deviation, and –log likelihood values obtained when achieving the maximum generalized *R*^2^ and entropy *R*^2^ in the training and validation sets.^25^

The predicted response values (*Y*) were calculated from each neuron in the hidden layer by summing each neuron and applying the weights as described in Eqs. (3) and (4).


(3) $$H_{i}=\mathrm{Tan}H\left(0.5\left(\sum_{m=1}^{N_{i}}(w_{i,m}x_{m})+b_{i}\right)\right)$$

(4) $$Y=\sum_{i=1}^{N_{h}}\left(w_{2,i}H_{i}\right)+b_{n}$$

where *H*_i_ is the hidden layer output; *N*_i_ and *N*_h_ represent the number of input neurons and the number of hidden layers, respectively; *w* is the connection weight; *x*_m_ is the corresponding value of the input variable; and *b*_i_ and *b*_h_ are the biases in the input and hidden layers, respectively.

Based on the relative weight predicted by the ANN model, the effect of each independent variable on the response was calculated using Eq. (5). The superscripts *i*, *h*, and *o* represent the input, hidden, and output layers of the proposed ANN model, respectively. The results are expressed as the percentage of importance *j* (*I*_j_).^25^


(5) Ij00=∑m=1m=Nhwjmjh/∑k=1k=Niwkmih×wmnho∑k=1k=Ni∑m=1m=Nhwkmih/∑k=1k=Niwkmjh×wmnho×100

###  Analysis of the dependent variables

####  Hydrodynamic diameter and zeta potential of NLCs

The hydrodynamic diameter, PDI, and zeta potential of the CFE-NLCs and blank NLCs were determined using a Zetasizer Nano ZS instrument coupled with Dispersion Technology software (version 5) (Malvern Instruments Ltd, Malvern, Worcestershire, UK).^23^ Before measurement, the samples were diluted with ultrapure water to a 1:300 dilution. All measurements were repeated at least three times at 25°C.

####  %EE

The %*EE* of 5CQA in the CFE-NLCs was determined by the ultrafiltration and centrifugation method. A sample (500 µL) was placed in a sample compartment with a filter membrane (molecular weight cutoff, 10 000 Da; Microcon YM-10, Sigma-Aldrich Company, USA) and centrifuged at 5000 rpm for 10 minutes at 4°C (Biofuge Fresco centrifuge, Heraeus Sepatech GmbH, Germany). The level of 5CQA in the filtrate was determined by the HPLC-PDA method (Section HPLC-PDA analysis). The %*EE* values of 5CQA in NLCs were calculated indirectly using Eq. (6).


(6) EE00=C1−C2C1×100

where *C*_1_ and *C*_2_ represent 5CQA in the CFE-NLC dispersion and supernatant, respectively.

###  Characterization of the optimized CFE-NLCs

####  Scanning electron microscopy (SEM)

The morphology of the NLCs was elucidated by SEM (S-3400 N, Hitachi, Japan).^23^ Each sample was diluted in deionized water, placed on carbon tape, and dried at room temperature. The samples were then gold-coated before analysis. SEM images were visualized under low vacuum at a 10 kV accelerating voltage.

####  Differential scanning calorimetry

The thermal behaviors of the CFE-NLCs, blank NLCs, and bulk raw materials were examined using a differential scanning calorimeter (DSC823^e^/400, Mettler-Toledo Corporation, Switzerland).^23^ Accurately weighed out freeze-dried samples and their ingredients (5–7 mg) were placed in an aluminum pan and analyzed by heating from 30°C to 100°C at 10°C/min while flushing with 40 mL/min N_2_. A hermetically sealed empty pan was used as a reference. DSC thermograms were then analyzed using STAR^®^ SW9.30 software.

####  Fourier transform infrared (FTIR) spectroscopy

The interaction between the CFE and the sample excipients was characterized by an FTIR spectrometer (Nicolet 6700, Thermo Fisher Scientific Inc., MA, USA).^23^ The spectra were measured between 4000 and 400 cm^–1^ with a transformation of 32 scan sizes and a spectral resolution of 4 cm^–1^. The results were analyzed using OMNIC software.

####  Release profile study

The release profiles of 5CQA in CFE-NLC and free CFE solutions were studied using a dialysis method. Samples (1 mL) (equivalent to 4000 µg of 5CQA) were poured into a preswollen dialysis bag (molecular weight cutoff, 12 000–14 000 Da; Sigma-Aldrich Company, St. Louis, MO, USA). The bag was dialyzed against phosphate-buffered saline (PBS;100 mL, pH 7.4) for 24 hours at 100 rpm and 37°C. The sample (1 mL) was collected at a predetermined time and replaced with an equivalent amount of fresh medium. All media contained 1% (*w/v*) polysorbate 80 to maintain sink conditions. The quantity of 5CQA was then analyzed by the HPLC-PDA method (Section HPLC-PDA analysis), and the result was expressed as the percentage of cumulative release. The possible release mechanisms according to the zero-order, first-order, Higuchi, Hixon-Crowell, and Korsmeyer-Peppas models were applied to fit the release kinetics.

####  Skin permeation study

The in vitro skin permeation analysis of CFE-NLCs was evaluated by Franz diffusion cells using free CFE dissolved in propylene glycol as a control group. Porcine ear skin was prepared as described in our previous study.^23^ Test samples (0.5 mL) were applied to the dorsal skin with an effective diffusion area of 1.86 cm^2^. The receiving chamber was filled with PBS (pH 7.4) containing 1% polysorbate 80 (12 mL) and maintained at 37°C with a surface temperature of 32°C under continuous stirring at 500 rpm. The receiver medium was withdrawn at 1, 3, 6, 12, and 24 hours. After 24 hours, the skin surface was washed with PBS. The stratum corneum was separated from the skin using tape stripping 15 times with 3M Scotch Tape (3M Thailand Ltd., Bangkok, Thailand). The 5CQA amounts in the tapes and the remaining chopped skin (viable epidermis and dermis) were extracted in a mixture of methanol and water (50:50 *v/v*) using a sonicator. The quantity of the extracted 5CQA was then determined by HPLC-PDA.

####  Hen’s egg test-chorioallantoic membrane (HET-CAM) test

The HET-CAM assay was applied to evaluate the irritation potential of CFE-NLCs according to the ICCVAM protocol.^26^ Fertilized hen eggs (Rhode Ireland Red) (weight between 50 and 60 g) were incubated for 9 days in an automated rotating machine (Nanchang Alex Electric Factory, Jiangxi, China) at 37.8 ± 0.3°C and 58.0 ± 2.0% relative humidity. The experiment was validated with 0.9% NaCl (negative control) and 0.1 N NaOH solution (positive control). The test solutions (300 µL) were then added to the CAM, and the irritation effect was observed under a stereomicroscope combined with Image Framework software (Nikon ZMZ 745T, Nikon Instruments Inc., Melville, NY, USA). The onset times in seconds of vascular hemorrhage, lysis, and coagulation were monitored within 300 s. The irritation scores were calculated according to Eq. (7):


(7) $$Irritation\;score=5\left(\frac{301-Hemorrhage}{300}\right)+7\left(\frac{301-Lysis}{300}\right)+9\left(\frac{301-Coagulation}{300}\right)$$

The irritation characteristics were rated on the following scale: from 0 to 0.9 as nonirritant; from 1 to 4.9 as mildly irritant; from 5 to 8.9 as moderately irritant; and from 9 to 21 as strongly irritant. The eggs were then frozen at −20°C overnight to kill the embryo at the end of the experiments.

####  Stability study

 The physicochemical stability of the CFE-NLCs was evaluated by storage at 4 ± 2°C, 25 ± 2°C, 40 ± 2°C, and 75 ± 3% relative humidity for 90 days.

###  Statistical analysis

All measurements were performed in at least triplicate, and the data obtained are expressed as the means ± standard deviations (SDs). The experimental results were analyzed by analysis of variance (ANOVA) followed by Tukey’s test at *P* < 0.05.

## Results and Discussion

###  Identification of polyphenols in CFE

The yield of CFE obtained by heat-reflux extraction was 16.90 ± 0.65 wt% (dry basis), and its phytochemicals were characterized by HPLC-PDA-ESI-MS. Several chlorogenic acid derivatives, which are compounds formed by ester bonds between quinic acid and certain transcinnamic acids, were detected in CFE. This result is attributed to the presence of fragmented ions [M–H]^–^ at *m/z* 353, 191, 179 and 135. The ultraviolet spectrum of the hydroxycinnamic moiety confirmed the identification, with maximum bands at approximately 242 nm and 320–329 nm and a shoulder (*sh*) at 295–300 nm.^16^

**Figure 1:**
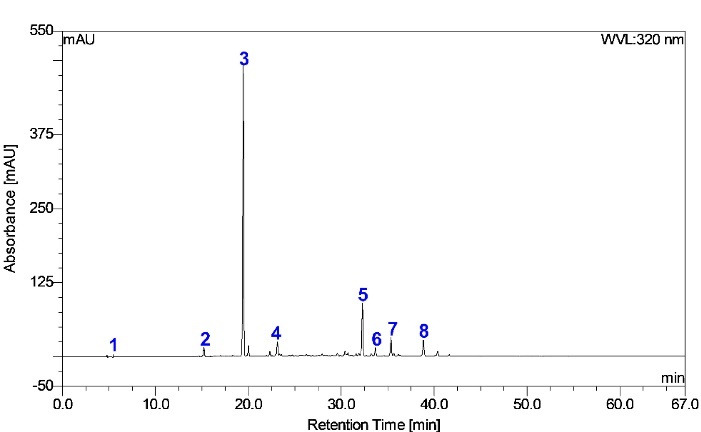
Typical HPLC chromatogram of CFE obtained at 320 nm

Peak **3** (Figure 1) (*t*_R_ 19.4 min, λ_max_ 247, 327, 296–298 nm) was predominant in the HPLC chromatogram. This peak revealed the spectral character of caffeoylquinic acid by showing the [M–H]^–^ parent ion at *m/z* 353 and [M–H]^–^ fragment ions at *m/z* 191 and 179. This fragmentation pattern is consistent with deprotonated quinic acid and caffeic acid.^16^ The potential MS^n^ fragmentation pathway of compound **3 **is detailed below. This substance showed deprotonated [M–H]^–^ ions at *m/z* 353 with cleavage of the entire B-ring, yielding a fragment ion at *m/z* 191 [M–162–H]^–^. Additional fragmentation of the precursor ion at *m/z* 191 triggers a loss of formic acid (CH_2_O_2_), opens the A-ring, and produces MS^3^ fragment ions at *m/z* 173 [M–180–H]^–^, *m/z* 127 [M–226–H]^–^ and *m/z* 85 [M–268–H]^–^. The MS^2^ base peak at *m/z* 191 with a weak MS^2^ ion at *m/z* 179 (<5% base peak) can be used to distinguish individual monoacyl chlorogenic acids. Thus, peak **3** was identified as 5CQA by comparison to the authentic standard and the literature.^13,16,27,28^

The above results agree well with the existing study on phytochemicals of CFE.^4^ In a previous study, ultrasound-assisted extraction was applied to prepare plant extracts, and only 5CQA was tentatively identified as the primary component.^4^ The details of substances found in small amounts are lacking. In contrast, CFE obtained by heat-reflux extraction was selected as an active ingredient for anti-skin-aging purposes in this work. The use of different extraction methods generally results in different qualities or quantities of botanical extracts.^4^ It is necessary to know both major and minor components before incorporating such plant extracts into NLCs. Therefore, the phytochemicals in CFE were further analyzed in more detail.

Other minor components were peaks **1**, **2**, **4**, **5**, **6**, **7**, and **8** (Figure 1; Table 3). Peak **1** showed an [M–H]^–^ ion at *m/z* 191 (*t*_R_ 3.41 min) (UV λ_max_ 198 nm); MS^2^ at *m/z* 127(100), 173(83), 85(71), and 93(48); and MS^3^ at *m/z* 85(100), 99(40), and 109(47). Thus, **1** was identified as quinic acid.^29^ Peak **2** (*t*_R_ 4.62 minutes, (λ_max_ 259, 285 and 323 nm) showed an [M–H]^–^ ion at *m/z* 371, and deprotonation of this compound yielded MS^2^ with a main fragment ion at *m/z* 353. The difference in mass between 371 and 353 is 18 Da, which can be explained by the addition of two hydrogen atoms and one oxygen atom to a chlorogenic moiety. The presence of fragment ions at *m/z* 353, 341, 191, 173, 161, 135, and 111 is indicative of CQA derivatives.^30^ Therefore, peak **2** was provisionally identified as dihydroxy-dihydro caffeoylquinic acid. Peak **4** (*t*_R_ 25 minutes) exhibited a molecular ion at *m/z* 337 and main fragment ions at *m/z* 191 [quinic acid–H]^–^ (UV λ_max_ 239 nm) and 173 [quinic acid–H-H_2_O]^–^ (UV λ_max_ 311 nm). These results indicate that the compound is a derivative of quinic acid or caffeic acid. Peak **4** was therefore tentatively defined as the *p*-coumaroylquinic acid isomer.^30^

**Table 3:** HPLC-PDA-ESI-MS data on the polyphenolic constituents in the aqueous ethanolic extract of CFE and proposed compounds

**Peak no.**	***t***_R_ ** (min)**	**UV λ**_max_ ** (nm)**	**[M–H]** ^–^ ***m/z***	**MS** ^n^	**Compound**
1	3.2	196, 211	191	MS^2^ (191): 127, 173, 85, 93; MS^3^ (127): 85, 99, 109, 127	Quinic acid
2	15.8	259, 285, 323	371	MS^2^ (371): 353, 341, 191, 173, 197: MS^3^ (353): 191, 179	Dihydroxydihydrocaffeoylquinic acid
3	19.4	247, 296-298*sh*, 327	353	MS^2^ (353): 191; MS^3^ (191): 127, 173, 111, 93, 85	5CQA
4	23.2	239, 311	337	MS^2^ (337): 191, 173; MS^3^ (191): 127, 173, 85	*p*-Coumaroylquinic acid isomer
5	32.7	240, 300*sh*, 326	515	MS^2^ (515): 353; MS^3^ (353): 191, 173, 179, 135	1,3-Dicaffeoylquinic acid (1,3-diCQA)
6	34.3	217, 240, 300*sh*, 327	515	MS^2^ (515): 353; MS^3^ (353): 191, 173, 179, 135	3,5-Dicaffeoylquinic acid (3,5-diCQA)
7	36.2	258, 298*sh*, 312	499	MS^2^ (499): 337, 353, 335, 173; MS^3^ (337): 173, 179, 191	4-*O*-*p*-coumaryl-5-*O*-caffeoylquinic acid
8	38.4	298*sh*, 313	483	MS^2^ (483): 337; MS^3^ (337): 173, 191, 99	4,5-di-*O*-p-coumaroylquinic acid (4,5-diCQA)

Peaks **5** (*t*_R_ 34.75 minutes, UV λ_max_ 240, 300 *sh*, 326 nm) and **6** (*t*_R_ 36.27 minutes, UV λ_max_ 240, 300 *sh*, 327 nm) showed both an [M–H]^–^ ion at *m/z* 515 and an [M–H]^+^ ion at *m/z* 517, indicating a molecular weight of 516. An [M–H]^–^ ion at *m/z* 515, fragment ion [M–C_9_H_6_O_3_]^–^ at *m/z* 353 (caffeoylquinic acid), and fragment ions at *m/z* 191 [M–H–2C_9_H_6_O_3_]^–^, *m/z* 173 [M–H–2C_9_H_6_O_3_–H_2_O]^–^, *m/z* 135 [M–C_7_H_10_O_5_–C_9_H_6_O_3_–COOH]^–^, and *m/z* 179 [M–H–C_9_H_6_O_3_–C_7_H_10_O_5_] were noted. These fragmentation patterns are consistent with the identification of the compound as dicaffeoylquinic acid (diCQA).^30^ The positions of the caffeic acid substituents in the 1,3-, 1,4- and 1,5-diCQA isomers could be distinguished using MS^n^ fragmentation.^27,28^ Thus, peak **5** was identified as 1,3-diCQA, whereas peak **6** was identified as 3,5-diCQA.^27-30^

Peaks **7** and **8** had similar UV spectra characteristic of a ferulic acid structure (UV λ_max_ 312–315 nm, 298 *sh* nm). Peak **7** showed a molecular ion at *m/z* 499. Deprotonation of this molecule produced a secondary ion at *m/z* 337. The base peak of MS^2^ (*m/z* 337) yielded *m/z* 173 in MS^3^. These results indicate that this compound was *vic*-4,5-*p*-coumaroyl-caffeoylquinic acid. The peak **7** fragmentation pattern was consistent with the previous report’s designation of 4-*O*-*p*-coumaryl-5-*O*-caffeoylquinic acid.^31^ Peak **8**, with an [M–H]^–^ ion at *m/z* 483, produced MS^2^ and MS^3^ base peaks at *m/z* 337 and *m/z* 173, respectively, due to the loss of a coumaroyl unit (146 Da). It was tentatively identified as 4,5-di-*O*-*p*-coumaroylquinic acid.^31^

 Overall, 5CQA was assigned as a functional and chemical marker for CFE because it was the vital component and exhibited the desired biological properties. The content of 5CQA, a significant phenolic compound determined by HPLC-PDA, was 241.66 ± 0.07 mg/g plant extract.

###  Biological properties of CFE

 Aging is not only a cosmetic concern but also a medical issue, as it is a complex process affecting the appearance, structure, mechanism, and function of the skin. Collagen production in naturally aged skin or skin aged by overexposure to ultraviolet radiation decreases with higher levels of MMPs. Reactive oxygen species levels can also be amplified by both intrinsic and extrinsic factors to stimulate mitogen-activating protein kinases. This mechanism can induce activator protein 1, promote the gene expression of MMPs and inhibit type I procollagen synthesis. Regulating cellular oxidative stress, collagen metabolism, and MMP levels is therefore an essential skin-care strategy. The tests below were performed to verify the in vitro effects of CFE for skin care applications.

####  HDFn cell viability

The cytotoxicity of CFE to HDFn cells was tested to determine the safe dose of plant extract. The MTT results showed that CFE had no toxic effects on HDFn at concentrations up to 1000 µg/mL, with cell viabilities of > 80%; thus, the IC_50_ of CFE could not be calculated from the experiment (Figure S1a). According to this result, CFE is considered a nontoxic substance ( > 80% cell viability indicates nontoxicity; 80-60% indicates weak toxicity; 60-40 indicates moderate toxicity; and <40% indicates high toxicity).^32^ Concentrations of plant extract of up to 100 µg/mL were used for further analysis of the biological properties of CFE.

####  Procollagen type I synthesis

Procollagen synthesis is a crucial factor that directly influences the regenerative process of the skin. To examine the effect of CFE on collagen synthesis, HDFn cells were treated with different concentrations of CFE, and the results are shown in Figure S1b. ELISA analysis revealed that all concentrations of CFE significantly increased type I procollagen synthesis compared with that in the untreated control group (*P* < 0.05). The effect was concentration dependent, as shown by the procollagen type I levels of 1268.05 ± 34.46, 1843.40 ± 53.74, and 2388.94 ± 45.69 pg/mL obtained after 48 hours of treatment with 1, 10, and 100 µg/mL CFE, respectively. CFE yielded significantly less stimulation than treatment with 200 µg/mL ˪-ascorbic acid (2893.23 ± 78.72 pg/mL), a positive control that is well known to promote procollagen production.

####  Inhibitory activity against MMPs

MMPs are a family of zinc-containing proteinases that cause extracellular matrix protein degradation. Increased production of MMP enzymes is not only linked with inflammation of human tissues but also associated with the degradation of collagen, which is related to natural aging and photoaging. In this study, extracellularly secreted MMP-1 (collagenase 1), MMP-3 (stromelysin 1), and MMP-9 (gelatinase B) were examined because the combined action of these enzymes can completely degrade collagen and components of the elastic network, leading to the formation of wrinkles in the skin. LPS-treated HDFn (1 µg/mL) increased the expression of MMP-1 (Figure S1c), MMP-3 (Figure S1d), and MMP-9 (Figure S1e) by more than twofold compared with that in untreated control cells (*P* < 0.05). Interestingly, MMP expression levels decreased significantly in a dose- and time-dependent manner compared with those in LPS-treated cells. CFE at doses of 1, 10 and 100 µg/mL markedly reduced MMP-1 production by 7.51 ± 0.22, 6.75 ± 0.16, and 6.46 ± 0.14 ng/mL at 12 hours of exposure, respectively, and 7.37 ± 0.24, 6.45 ± 0.25, and 6.24 ± 0.27 ng/mL at 24 hours of treatment, respectively. A similar trend was also observed for MMP-3 and MMP-9 levels: higher doses corresponded to greater potency. A possible explanation could be that polyphenols in CFE can chelate metal ions, decreasing the free zinc concentration needed for MMPs.

These results provide the first evidence that CFE potentially exerts an antiaging effect by promoting collagen synthesis and suppressing the production of MMPs to minimize the formation of skin wrinkles. The literature review also indicated that CFE could be used as an antiaging agent due to its cellular protective effects against oxidative stress and anti-inflammatory properties.^4,7^ Furthermore, 5CQA, a principal metabolite in CFE, has exceptional biological activities for skin antiaging. This active molecule can promote type 17 collagen production, protect against ultraviolet A-induced oxidative stress, exert anti-inflammatory activities, and prevent anionic surfactant-induced epidermal itching.^8-11^ As a result, CFE could be used as a cosmetic antiaging ingredient.

###  Model fitting by the ANN algorithm

A summary of the 30 experimental runs performed to study the influence of the parameters on the physicochemical properties of the resulting NLCs is given in Table 2. The ranges of *Y*_1_, *Y*_2_, *Y*_3_, and *Y*_4_ were 53.25–489.33 nm, 0.03–0.45, –5.76–14.89 mV, and 40.30–80.76%, respectively. The ANN algorithm was implemented in this study due to its ability to model nonlinear relationships between variables with reasonable predictability.^25^

**Figure 2:**
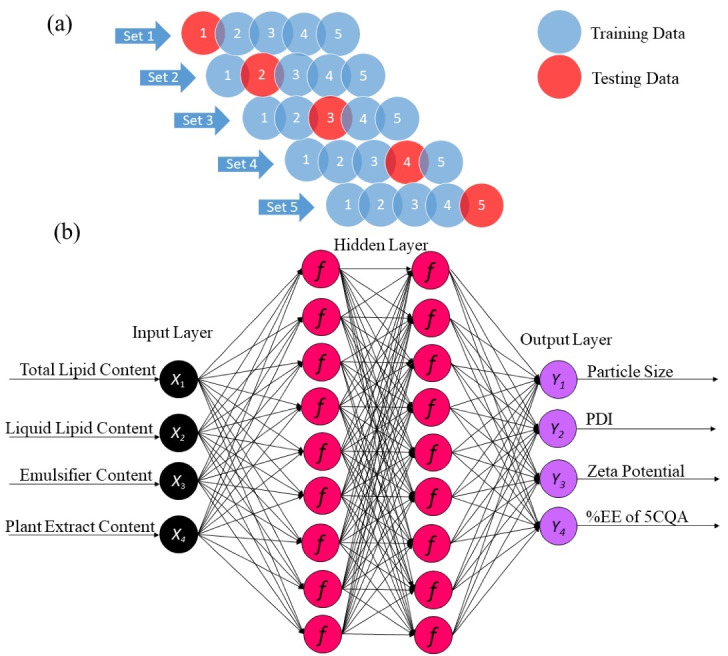
(**a**) Schematic diagram of the K-fold cross-validation algorithm (K = 5) applied to the datasets. (**b**) Diagram of the ANN architecture adopted for modeling and optimization of the CFE-NLCs in this study

Several cross-validation methods have been implemented for ANN modeling, including the jackknife test (K-fold and leave-one-out), bootstrapping, the Monte Carlo test, the three-way split test, and the disjoint set test. This study applied K-fold cross-validation to run the algorithm. Unlike the three-way split test, the K-fold method (K = 5) contained only two datasets; random division into 80% for training and validation and 20% for testing subgroups was performed in our analysis (Figure 2a). The presented method with the *TanH* optimizer created the best architecture of the ANN, which is advantageous. A 4-9-9-4 topology of input, hidden, hidden, and output layers, respectively (Figure 2b), was chosen as the best ANN model due to its high predictive performance (Table 4). The correlations between the experimental values and the predicted values of all the responses in the training and validation sets were plotted (Figure S2). The experimental values of the responses were fitted to a straight line with a determination coefficient (*R*^2^) > 0.82. In addition, low and randomly dispersed residues were observed for both datasets, revealing that the errors were not correlated and had the same variances. These results suggested that the derived mathematical equations were acceptable and could be used to predict the response variables in the design space. The obtained hidden layer (*H*) codes are described by Eq. (8):

*H*_1_ = *TanH*(0.5(0.69*X*_1_ – 0.09*X*_2_ – 0.28*X*_3_ – 2.79*X*_4_ + 3.07));

*H*_2_ = *TanH*(0.5(–0.66*X*_1_ – 0.26*X*_2_ – 1.99*X*_3_ –0.62*X*_4_ + 18.45));

*H*_3_ = *TanH*(0.5(–0.24*X*_1_ + 0.07*X*_2_ – 0.07*X*_3_ – 2.57*X*_4_ + 0.50));

*H*_4_ = *TanH*(0.5(0.22*X*_1_ + 0.26*X*_2_ – 0.29*X*_3_ – 1.00*X*_4_ – 4.35));

*H*_5_ = *TanH*(0.5(0.05*X*_1_ – 0.22*X*_2_ + 0.74*X*_3_ + 2.24*X*_4_ + 1.08));

*H*_6_ = *TanH*(0.5(0.09*X*_1_ + 0.05*X*_2_ – 1.88*X*_3_ + 4.23*X*_4_ – 2.99));

*H*_7_ = *TanH*(0.5(0.05*X*_1_ – 0.14*X*_2_ – 1.91*X*_3_ + 2.81*X*_4_ + 7.28));

*H*_8_ = *TanH*(0.5(–1.22*X*_1_ + 0.13*X*_2_ + 0.66*X*_3_ + 0.01*X*_4_ + 2.31));

*H*_9_ = *TanH*(0.5(–0.92*X*_1_ – 0.08*X*_2_ – 1.42*X*_3_ – 2.40*X*_4 _+ 14.92));

*HH*_1_ = *TanH*(0.5(–2.47*H*_1_ + 1.80*H*_2_ – 1.04*H*_3_ + 0.17*H*_4_ + 2.64*H*_5_ + 2.81*H*_6_ + 2.45*H*_7_ + 0.76*H*_8_ – 4.03*H*_9_ + 1.97));

*HH*_2_ = *TanH*(0.5(–0.58*H*_1_ + 3.96*H*_2_ – 0.95*H*_3_ + 1.12*H*_4_ – 2.31*H*_5_ – 2.05*H*_6_ + 1.00*H*_7_ – 1.42*H*_8_ + 0.14*H*_9_ – 0.61));

*HH*_3_ = *TanH*(0.5(–0.55*H*_1_ + 2.30*H*_2_ + 0.51*H*_3_ + 0.31*H*_4_ – 1.62*H*_5_ + 0.14*H*_6_ + 0.49*H*_7_ – 0.77*H*_8_ + 4.15*H*_9_ –1.34));

*HH*_4_ = *TanH*(0.5(0.81*H*_1_ – 0.25*H*_2_ + 0.48*H*_3_ – 5.18*H*_4_ + 0.60*H*_5_ – 0.70*H*_6_ – 0.78*H*_7_ + 1.88*H*_8_ + 2.67*H*_9_ + 0.34));

*HH*_5_ = *TanH*(0.5(2.41*H*_1_ – 1.86*H*_2_ – 1.43*H*_3_ + 1.12*H*_4_ – 1.49*H*_5_ – 0.55*H*_6_ + 3.74*H*_7_ – 1.08*H*_8_ – 0.14*H*_9_ + 2.29));

*HH*_6_ = *TanH*(0.5(0.12*H*_1_ + 2.43*H*_2_ – 1.94*H*_3_ – 0.92*H*_4_ –1.63*H*_5_ – 2.39*H*_6_ + 1.14*H*_7_ – 1.90*H*_8_ + 0.50*H*_9_ – 0.97));

*HH*_7_ = *TanH*(0.5(–0.64*H*_1_ – 0.17*H*_2_ – 1.41*H*_3_ – 0.68*H*_4_ + 0.32*H*_5_ – 2.35*H*_6_ + 0.73*H*_7_ – 1.95*H*_8_ – 0.18*H*_9_ – 0.92));

*HH*_8_ = *TanH*(0.5 (0.10*H*_1_ + 0.87*H*_2_ – 1.34*H*_3_ – 1.35*H*_4_ – 0.56*H*_5_ – 3.26*H*_6_ + 1.15*H*_7_ – 0.77*H*_8_ – 0.94*H*_9_ – 0.82)); and

*HH*_9_ = *TanH*(0.5(–1.09*H*_1_ – 2.39*H*_2_ – 2.54*H*_3_ – 0.35*H*_4_ + 0.40*H*_5_ – 1.37*H*_6_ – 0.10*H*_7_ + 0.05*H*_8_ + 1.26*H*_9_ – 1.94))

 (8)

 The relationship between the responses of the CFE-NLCs and the tested independent variables is shown by the final layer codes (Eqs. 9–12).

*Y*_1_ (nm) = 18.19*HH*_1_ – 40.03*HH*_2_ + 26.22*HH*_3_ – 146.97*HH*_4_ – 78.91*HH*_5_ + 180.00*HH*_6_ + 121.12*HH*_7_ – 112.00*HH*_8_ – 102.29*HH*_9_ + 170.31;

 (9)

*Y*_2_ = –0.02*HH*_1_ + 0.09*HH*_2_ – 0.04*HH*_3_ – 0.07*HH*_4_ – 0.10*HH*_5_ + 0.20*HH*_6_ + 0.03*HH*_7_ – 0.13*HH*_8_ + 0.13*HH*_9_ + 0.30;

 (10)

*Y*_3_ (mV) = –2.54*HH*_1_ + 0.15*HH*_2_ – 1.86*HH*_3_ + 0.35*HH*_4_ + 0.94*HH*_5_ + 2.50*HH*_6_ – 0.33*HH*_7_ – 0.10*HH*_8_ + 3.16*HH*_9_ – 10.73; and

 (11)

*Y*_4_ (%) = 3.90*HH*_1_ + 29.57*HH*_2_ – 10.98*HH*_3_ – 1.71*HH*_4_ – 5.88*HH*_5_ – 18.64*HH*_6_ – 12.30*HH*_7_ + 14.02*HH*_8_ + 11.59*HH*_9_ + 61.43

 (12)

**Table 4:** Summary of statistical indicators for the developed ANN model obtained from a 4-9-9-4 architecture

**Response**	**Statistical indicator**	**Training set**	**Testing set**
	**Measures**	**Values**	**Values**
*Y*_1_	*R* ^2^	0.9731	0.9994
	RMSE	20.3926	1.3689
	Mean absolute deviation	7.8443	0.7518
	-Log-likelihood	106.4187	10.3981
	Error sum of squares	9980.6204	11.2448
	Sum frequency	24.0000	6.0000
*Y*_2_	*R* ^2^	0.9448	0.9557
	RMSE	0.0187	0.0159
	Mean absolute deviation	0.0137	0.0112
	-Log-likelihood	-61.4794	-16.3259
	Error sum of squares	0.0084	0.0015
	Sum frequency	24.0000	6.0000
*Y*_3_	*R* ^2^	0.9114	0.9819
	RMSE	0.7047	0.3213
	Mean absolute deviation	0.4319	0.2997
	-Log-likelihood	25.6564	1.7013
	Error sum of squares	11.9199	0.6194
	Sum frequency	24.0000	6.0000
*Y*_4_	*R* ^2^	0.8257	0.9987
	RMSE	3.9761	0.4609
	Mean absolute deviation	1.8689	0.2314
	-Log-likelihood	67.1818	3.8659
	Error sum of squares	379.4265	1.2744
	Sum frequency	24.0000	6.0000
Summary	Generalized *R*^2^	1.0000	1.0000
	-Log-likelihood	193.8338	8.2782

Overfitting of a built ANN model may occur when any learning system matches its training set almost perfectly but continually struggles with unseen data.^33^ In this study, K-fold cross-validation strengthened the overfitting network. By running the algorithm, the initial datasets were randomly split into training and testing subgroups. K rounds (an appropriate K of 5 was selected in this study) of training-validation-testing were performed on different, nonoverlapping, and equally proportioned training, validation and testing sets. This strategy could mitigate errors in applications with unseen data, as supported elsewhere.

Overall, the applied computerized modeling approach was successful in fitting the NLC model. The ANN approach used in this study has a number of advantages. This algorithm effectively handles a large number of variables with varying degrees of complexity by generating a solution with a high predictive accuracy. It is capable of completing tasks that are inaccessible to nonlinear RSM models. Due to its parallel nature, ANN technology produces high-quality output even in the presence of noise and can continue operating uninterrupted even if the network fails. However, ANNs have some drawbacks in that they require a large amount of data for trial and error to emulate the appropriate architecture. As a result of this feature, the training data required a lengthy processing period.^24,25^

###  Influence of independent variables on the responses of NLCs

In this study, the experimental design was limited to four formulation variables: the proportions of solid lipids, liquid lipids, surfactant, and plant extract. These parameters and their associated ranges were chosen in light of our preliminary findings.^22^ Other process parameters were kept constant throughout the trials to avoid interference. Setting the temperature of the oil and aqueous phases to 80°C was sufficient to melt the solid lipids without decomposition of 5CQA in CFE during formulation preparation. This temperature produced desired droplets of coarse oil in the water emulsion. Another factor to consider is the particle size reduction process for NLCs using high-speed homogenization and probe sonication methods. The prepared nanoparticles should have a particle size of less than 500 nm to be delivered to the skin.^23^ Both methods produce a proper length, which contributes to good NLC quality. The particle size reduction time of 10 minutes obtained using a high-speed homogenizer resulted in a satisfactory coarse emulsion. Additionally, when a probe sonicator was used for 10 minutes, a sufficiently fine stable emulsion in the nanometer range was produced. Beyond this time limit, the particle size of NLCs could not be reduced further.

The *I*_j_ of each input variable with respect to the output was calculated based on the weights and biases of each neuron in the hidden layer of the developed ANN models.^25^ As shown in Figure S3, all the factors studied affected the properties of the NLCs. The proportion of emulsifiers (*X*_3_) was the main factor influencing the size of the nanoparticles (*I*_j_ = 67.70%), followed by the liquid lipid content (*X*_2_) (*I*_j_ = 42.60%), solid lipid content (*X*_1_) (*I*_j_ = 41.80%), and CFE content (*X*_4_) (*I*_j_ = 8.10%). The particle size distribution of CFE-NLCs was highly affected by the proportion of liquid lipids (*X*_2_) (*I*_j_ = 67.80%), while the proportion of emulsifiers had the greatest effect on the zeta potential (*I*_j_ = 68.80%). A similar pattern of *I*_j_ for each input factor was observed for the response of both particle size and 5CQA %EE (*X*_1_, *I*_J_ = 52.30%; *X*_2_, *I*_J_ = 56.70%; *X*_3_, *I*_J_ = 59.6%; *X*_4_; *I*_J_ = 38.80%). Overall, the influence of each factor on all responses could be ranked from highest to lowest as follows: *X*_3_ (59.70%) > *X*_2_ (49.50%) > *X*_1_ (42.10%) > *X*_4_ (32.10%).

 Three-dimensional (3D) surface plots were then constructed from the nonlinear models given in Eqs. (8)–(12) to understand the interactive relationships between each factor and the dependent variables. Each plot of two independent variables affected the responses of the CFE-NLCs. The remaining independent variables were fixed to be constant at zero.

####  Particle size of CFE-NLCs

**Figure 3:**
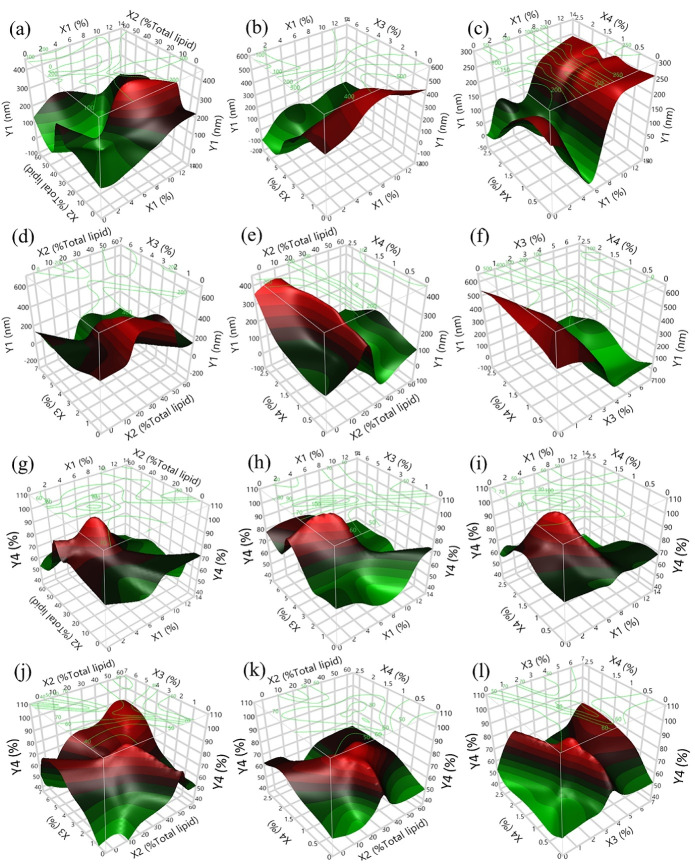
3D plots showing the combined effects of independent variables on the hydrodynamic diameter (**a**–**f**) and %EE of 5CQA in NLCs (**g**–**l**): (**a**, **g**) *X*_1_ versus *X*_2_; (**b**, **h**) *X*_1_ versus *X*_3_; (**c**, **i**) *X*_1_ versus *X*_4_; (**d**, **j**) *X*_2_ versus *X*_3_; (**e**, **k**) *X*_2_ versus *X*_4_; and (**f**, **l**) *X*_3_ versus *X*_4_

Particle size and size distribution are key factors affecting the quality of nanoparticles.^20^ Enhanced bioavailability of active ingredients is expected in the presence of nanosized carriers compared to that in the raw complex matrix. The composition of the formulation significantly affected the particle size of CFE-NLCs (*P* < 0.05). As demonstrated in Figure 3a−f, the proportion of emulsifiers (*X*_3_) was the parameter that most strongly affected the particle size of the CFE-NLCs, followed by the liquid lipid content (*X*_2_) and solid lipid content (*X*_1_). This impact can be attributed to the ability of emulsifiers to reduce interfacial tension by adsorbing at the oil-water interface and creating a tightly packed surfactant film.^34^ Increasing the solid lipid content above 6.5 wt% increased the size of the nanoparticles (Figure 3a–c). This finding is consistent with a previously reported result.^34^ The solid lipid content of the NLCs should not be greater than 6.5 wt% to achieve an average particle size within the nanometer scale (usually within the 1–100 nm range). Qadir et al^35^ stated that with a total lipid content of 3.2 wt% and a surfactant mixture content of 1.6 wt%, optimum NLCs coencapsulating *Smilax china* and *Salix alba* methanolic extracts could be achieved. In addition, appropriate polyphenol-rich *Hibiscus sabdariffa* extract-loaded NLCs consisted of 2.21 wt% total lipids and 1.93 wt% surfactant. On the other hand, a negative effect was observed in the case of the *X*_2_ and *X*_3_ variables, meaning that smaller NLCs were obtained at higher concentrations of liquid lipid and emulsifier (Figure 3a, b, d, e, f). However, the concentrations of CFE in the studied range (0.13–2.63 wt%) did not influence the particle size of CFE-NLCs (Figure 3c, e, f).

####  PDI of CFE-NLCs

PDI can be used to assess the distribution of the particle size of CFE-NLCs (Figure S4). This index has a value between 0 and 1; a value less than 0.3 indicates high homogeneity.^20^ PDI was profoundly impacted by the proportion of liquid lipids (*X*_2_) (67.80%) and exhibited a decreasing trend when the input value was greater than 29% of the total lipid content (Figure S4a, d, e). This phenomenon was attributed to the vigorous homogenization: cavitation detached nanoemulsion droplets with a high content of liquid lipids and therefore softer structures than droplets with a lower content of liquid lipids, which were more rigid. Other researchers reported a similar finding.^34^ Similarly, a proportion of emulsifier greater than 3.30 wt% resulted in a low PDI of nanoparticles because such proportions were adequate to stabilize the newly formed fine droplets (Figure S4b, d, f). Nevertheless, the PDI increased when the proportion of solid lipids was greater than 6 wt% (Figure S4a–c) because the high density of the nanoemulsion droplets hampered the ability of the high-speed homogenizer and ultrasound to disrupt them in the particle size reduction process.

####  Zeta potential of CFE-NLCs

Zeta potential is a measurement of the surface charge at the Stern layer of nanoparticles in a colloidal solution and can predict the stability of colloidal systems.^17^ Theoretically, long-term stability can be expected for nanoparticles with zeta potential values greater than ± 25 mV (0 to ± 5 = flocculation or coagulation stability behavior, ± 10 to ± 30 = incipient instability, ± 30 to ± 40 mV = moderate stability, ± 40 to ± 60 mV = good stability, and greater than ± 60 = excellent stability).^36^ However, there are still exceptions for some nanoparticles prepared using nonionic surfactants with low or no surface charge; the delivery system can be stabilized with a steric effect derived from the side chain of the surfactant used.^37^ The role of poloxamer 188 in preventing eventual agglomeration due to interparticle interactions, including van der Waals interactions, hydrophobic interactions, and hydrogen bonding, has been reported elsewhere.^23^

The zeta potential of the CFE-NLCs was found to be between –5.79 mV and –14.87 mV (Figure S2). Increasing the proportion of emulsifier in the formulation above 4.5 wt% resulted in a lower zeta potential of almost zero (Figure S5b, d, f), which was consistent with the effect of total lipid content (Figure S5a−b). A more negative zeta potential value was obtained by increasing the proportions of liquid lipids and CFE (Figure S5c−f). This negative surface charge was not attributed to the nonionic surfactant used but might be linked to free fatty acids or the oil-water interface adsorption of OH^-1^ water species and ionized phenolic acids derived from CFE.

####  %EE of 5CQA in NLCs

The effects of the input factors on the 5CQA %EE in CFE-NLCs are demonstrated in Figure 3g–l. The %EE of 5CQA ranged from 40.30−80.76%. This result could be attributed to some 5CQA dispersed in a free form in the colloidal solutions. The proportions of liquid lipids (*X*_2_) and emulsifier (*X*_3_) showed a highly nonlinear effect on the encapsulation of the target compounds. The encapsulation of 5CQA increased rapidly in the presence of liquid lipids and emulsifier at approximately 30% to 40% of total lipids and 3 wt% to 4 wt%, respectively (Figure 3g−l). In contrast, more than 7 wt% *X*_1_ decreased the %EE (Figure 3g−i). This result probably occurred because the 5CQA in CFE is not highly lipophilic. Therefore, a high proportion of lipid content may not be appropriate for the encapsulation of such substances.

Ideally, a high loading amount of CFE in the formulation is desired; however, increasing the concentration of *X*_4_ to greater than 1.70 wt% gradually decreased the %EE of 5CQA in NLCs. This result could be explained by the fact that excessive 5CQA cannot disperse in the NLC system. Medium levels of input factors are favorable for high %EE. These results are consistent with those reported previously by researchers.^38^ Therefore, *X*_1_, *X*_2_, *X*_3_, and *X*_4_ were very significant parameters influencing %EE.

###  Optimization and verification of the CFE-NLCs

The target NLCs should have a minimized particle size, good homogeneity, and low zeta potential, whereas the %EE of 5CQA should be maximized (Table 1). A desirability function was applied to this multiobjective optimization.^23^ The optimal CFE-NLCs (desirability value = 85.77%) should be composed of 4.80 wt% *X*_1_, 30.00% *X*_2_ (total lipids, equivalent to 1.44 wt% liquid lipids), 3.50 wt% *X*_3_, and 1.65 wt% *X*_4_. Three batches of CFE-NLCs were then prepared to confirm the model accuracy. There were no statistically significant differences between the predicted values (*Y*_1_ = 61.87 ± 22.38 nm, *Y*_2_ = 0.19 ± 0.03, *Y*_3_ = –10.85 ± 0.51 mV, and *Y*_4_ = 85.49 ± 3.82%) and the experimental values (*P* > 0.05) (*Y*_1_ = 63.32 ± 4.22 nm, *Y*_2_ = 0.17 ± 0.03, *Y*_3_ = –11.18 ± 0.36 mV, and *Y*_4_ = 84.72 ± 0.20%). These results suggest that the predicted ANN models are not biased compared with the experimented values. The developed CFE-NLCs showed a mean hydrodynamic diameter of 63.32 ± 4.22 nm. The small size of the nanoparticles should play an important role in promoting good stability because the particles remain in Brownian motion, which prevents sedimentation.^34^ The zeta potential value of the CFE-NLC dispersion was –11.20 ± 0.40 mV. These results suggest that electrostatic force is one of the mechanisms used to resist flocculation and sedimentation of the nanoparticles, along with a steric hindrance effect.^36^ This property plays an important role in the long-term stability of nanoparticles.

The %*EE* of 5CQA in NLCs was 84.72 ± 0.20%, indicating that a high amount of this substance was encapsulated in the NLCs, while the remaining 5CQA was available in the continuous phase. The trapping of polyphenols derived from *Hibiscus sabdariffa* extract by NLCs showed results similar to our findings.^38^ Similar findings were also reported in previous research: the %*EE* of green robusta coffee bean extract in NLCs was approximately 73%.^15^ These results may have occurred because certain polyphenols are not encapsulated in NLCs but combine with surfactants to form micelles.^39^ This unencapsulated 5CQA is expected to be released immediately at the site of action, which is a property of a good sustained delivery system.

###  Characterization of the optimal CFE-NLCs

####  Morphology of CFE-NLCs

The physical characteristics of CFE-NLCs are shown in Figure S6. The addition of CFE to the NLCs resulted in a change from opaque white to pale yellow (Figure S6a). The resulting CFE-NLCs had a hydrodynamic diameter between 28 and 142 nm (63.32 ± 4.22 nm) with good reproducibility (Figure S6b). SEM was used to analyze the microstructure of the CFE-NLCs. The morphology of CFE-NLCs (Figure S6c) and blank NLCs (Figure S6d) showed a spherical shape with a narrow size distribution and good dispersion. The particle sizes of the formulations established were close to 65 nm, which is similar to the values determined by photon correlation spectroscopy (Figure S6b). The small difference between the particle sizes determined by the two methods can be attributed to the differences in the sample preparation process and the measurement principle. A spherical shape of developed NLCs consisting of different formulation compositions was also documented in previous studies.^17,20^

####  Thermal behavior

DSC analysis was employed to investigate the thermal behavior of the CFE-NLCs and their excipients. The thermograms in Figure S7 show that glyceryl behenate, a physical mixture of glyceryl behenate and glyceryl monostearate (1:1), glyceryl monostearate, and poloxamer 188 had melting temperature peaks at 77.47°C, 74.53°C, 65.50°C, and 60.18°C, respectively. The onset time, end time, and melting temperatures of the blank NLCs and CFE-NLCs decreased compared to those of the corresponding bulk materials. The melting curves of the blank NLCs displayed three endothermic peaks at 47.24°C, 71.28°C, and 71.78°C, which indicated that the melting temperature of the solid lipids decreased when NLCs were formulated because the NLCs had less of a crystalline lattice than the bulk materials.^20^

The addition of CFE to the NLCs reduced the melting temperature peaks to 42.28°C, 55.92°C, and 63.59°C. The lower melting point of the CFE-NLCs than that of the blank NLCs can be ascribed to the dispersion of CFE in the lipid matrix of the nanoparticles. A minor endothermal peak for CFE at 71.28°C was also observed, but this peak disappeared when CFE was loaded into the NLCs. This result can be attributed to the almost complete dissolution of plant extract in the developed carriers or to the solubilization of the plant extract in the lipid matrix when the sample was heated.^40^ In addition, the CFE-NLCs showed no sharp peak, indicating a heterogeneous crystalline structure in the nanoparticles. The onset and melting temperatures of both CFE-NLCs and empty NLCs remained above 40°C, indicating that the formulations formed were in a solid state at human body temperature (37°C). The fabricated nanoparticles need to be in a solid state at body temperature to boost the stability of the delivery system and regulate the release of the active ingredient, as described elsewhere.^40^

####  FTIR spectroscopy

Interactions between the CFE and the formulation excipients were investigated using FTIR, and the results are shown in Figure S8. The fingerprint of the CFE displayed absorption bands at 3300 cm^–1^ and 2900 cm^–1^, representing the stretching of intramolecular O-H and C-H bonds, respectively, in aromatic compounds. The bands at approximately 1700 cm^–1^ and 859 cm^–1^) correspond to C = O stretching vibrations and OH-H bending vibrations, respectively, of carboxylic acid groups. C = C stretching in aromatic compounds is represented by the band at 1600 cm^–1^. The absorption band of CH_3_ (methylene groups) is located at 1398 cm^–1^, and the C-O vibrations of phenolic alcohols correspond to the bands at 1180 cm^–1^ and 950 cm^–1^. The band at 709 cm^–1^ represents the C-H stretching of aromatic compounds.

Concerning the CFE-NLC spectrum, the most significant changes observed in the fingerprint region indicated that CFE was present in the lipid matrix. Owing to the C-H and C = O vibrations of the lipids used in the formulation, the absorption band showed stronger signals at 2900 cm^–1^ and 1700 cm^–1^. No shifts in valency vibrations or new peaks appeared, indicating no chemical modification of the nanocarriers. The spectrum of the CFE-NLCs was almost the same as that of the blank NLCs. This finding is also consistent with the results of other studies showing that the active ingredient is trapped inside NLCs without chemically interacting with the formulation excipients.^17^

####  Release study

The release profiles of 5CQA from CFE-NLCs against PBS (pH 7.4) for 24 hours at 37°C are shown in Figure S9a. 5CQA in the free CFE solution was entirely released within 3 hours. In contrast, 5CQA from NLCs showed a biphasic release pattern beginning with a burst release at the initial stage, followed by a slower sustained release phase. In the first two hours, 19.74 ± 1.21% 5CQA was released from the CFE-NLCs. These phenomena should be attributed to the release of 5CQA-enriched NLC shells, 5CQA solubilized in surfactant micelles, and free 5CQA molecules.^20^ 5CQA showed a sustained release rate between 3 hours (22.58 ± 3.24%) and 24 hours (61.12 ± 3.17%). This occurrence should be due to the diffusion of 5CQA from the core of the NLC particles to the outer aqueous solution.

The chemical marker showed a biphasic release pattern due to the difference in melting temperature between the solid lipids (glyceryl behenate+glyceryl monostearate) and liquid lipids (caprylic/capric triglyceride). Solid lipids have a higher melting temperature, leading to the initial crystallization of particles with little liquid lipids or a liquid lipid-free core. Liquid lipids, which are mobile liquids at room temperature, are deposited on the particles’ outer shell, leading to the formation of a phenolic compound-enriched shell. As a result, the loading of CFEs into NLCs could be used to accelerate the onset of action of their anti-skin-aging properties, followed by a continued release period.^34^ Various mathematical models were then applied to study the kinetic release of 5CQA from CFE-NLCs. The results showed that 5CQA release had *R*^2^ values of 0.9926, 0.9660, 0.9541, 0.9335, and 0.8820 when fitted to the Higuchi, Korsmeyer-Peppas, first-order, Hixon-Crowell, and zero-order models, respectively. These release kinetics are most consistent with the Higuchi model, indicating that this bioactive molecule is released from NLCs by dissolution and diffusion mechanisms.

####  Skin retention study

The skin permeation profiles of active ingredients from CFE-NLCs and free CFE dissolved in propylene glycol were quantitatively analyzed using Franz diffusion cells. The results showed that the quantity of 5CQA that permeated through the porcine ear skin to the receptor medium was not detectable after 24 hours of incubation of the formulations. The tape stripping technique was used to isolate phytochemicals in the stratum corneum, and the retained 5CQA was extracted from the remaining skin (both viable epidermis and dermis). Figure S9b shows that the 5CQA from CFE-NLCs was localized mainly in the stratum corneum layer. The CFE-NLCs displayed 143.21 ± 7.98 µg/cm^2^ accumulation of 5CQA in the stratum corneum, while that in the viable epidermis and dermis was 17.12 ± 2.31 µg/cm^2^. This result suggests that the developed nanocarriers might have formed drug depots in the stratum corneum. The skin permeation into the viable epidermis and dermis layer was 2.31-fold greater than that of the free drug solution. This phenomenon occurred likely due to the small particle size and occlusive effect of the developed nanoparticles. Concerning the control group, propylene glycol can act as a permeation enhancer by solubilizing keratin in the stratum corneum, thus improving the permeation of 5CQA. CFE-NLCs facilitate the absorption of active compounds by the skin with minimal systemic loss and may promote the bioavailability of CFE. The results from this study and previously published data support this conclusion.^21^

####  Cytotoxicity study

In Figure S9c, the cell viability percentages of HDFn are plotted as a function of the concentrations of the CFE-NLCs and blank NLCs. At a concentration of 5000 µg/mL NLCs, which corresponded to 92 μg/mL CFE inNLCs, both tested nanoparticles exhibited mild toxicity to the test cells (cell viability > 80%). In contrast, doxorubicin HCl (10 μM), a positive control, significantly reduced cell viability to 31.41 ± 0.45% (*P* < 0.001). Several factors can influence the cytotoxicity of NLCs, including nanocarrier particle size and the nature of the liquid lipids, solid lipids, and nonionic surfactants. In this study, the lipids used in NLC formulations are well recognized as generally recognized as safe (GRAS) substances, and their ingredients are intended for topical application. These excipients could be considered to be highly biocompatible, as supported by other researchers.^21^

####  Irritation potential

**Figure 4:**
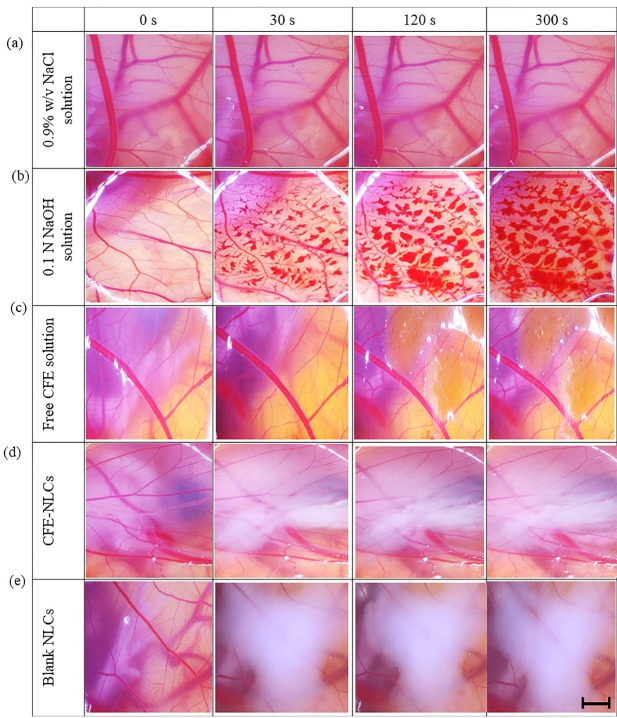
Images of HET-CAM incubated in direct contact with the test substances for between 0 and 300 s: (**a**) 0.9% w/v NaCl solution, (**b**) 0.1 N NaOH solution, (**c**) free CFE solution, (**d**) CFE-NLCs, and (**e**) blank NLCs. The scale bar is 1 mm (*n* = 6)

The HET-CAM test, an alternative model to animal experiments, was used to assess the potential for irritation of CFE-NLCs against vascular toxicity. NaCl solution (0.9% w/v) caused no change in the membrane (irritation score = 0.00 ± 0.00), while 0.1 N NaOH solution caused severe irritation (19.49 ± 0.46) (*P* < 0.001) (Figure 4a−b). The positive control solution caused major damage to the CAM with bleeding, lysis of blood vessels, and rosette-like coagulation. These results indicate that the approach implemented in this study is valid. Free CFE solution (1.65 mg/mL) exhibited an irritation score of 3.19 ± 0.49, indicating mild irritation (Figure 4c). The blank NLCs (2.03 ± 0.25) displayed an irritation score equivalent to that of the CFE-NLCs (1.21 ± 0.86) (*P* > 0.05) (Figure 4d−e). This outcome indicates that encapsulating CFE in those nanocarriers reduces its irritation potential compared with that of free plant extract solution (*P* < 0.05). A possible reason is the high %EE of 5CQA and controlled release behavior of NLCs, resulting in less irritation in the HET-CAM assay. Thus, CFE-NLCs are mild, tolerable irritants and therefore suitable for the skin.

####  Stability of the CFE-NLCs

One of the key findings of this study was that the optimized NLCs could improve the physicochemical stability of CFE. As seen in Figure S10a−g, CFE-NLCs were stable after storage at 4 ± 2°C and 25 ± 2°C for up to 90 days, suggesting satisfactory stability of the formed colloidal system. At 90 days of storage at 40 ± 2°C, minor changes in some properties of the CFE-NLCs, including the particle size, PDI, zeta potential, and %EE of the active compound, were observed (*P* < 0.05). A plausible and useful theory attributes the small loss of trapping capacity of 5CQA to the repulsion of encapsulated substances to the surrounding aqueous phase during storage.

Concerning chemical stability, there was no alteration in the content of 5CQA in either CFE-NLCs or free CFE for up to 90 days (*P* > 0.05). Nitthikan et al^15^ reported similar observations in their experiments. NLCs containing green robusta coffee bean extract exhibited stable chemical and DPPH^•^ free radical scavenging activities when the formulations were stored at 4°C and 30°C for 90 days. This finding is attributed to chemical deterioration. NLCs could help reduce the loss of 5CQA in the formulation and enhance the water solubility of CFE. In addition to indicating the value of this plant extract, the results obtained may lead to the use of other botanical extracts containing high 5CQA levels.

## Conclusion

 CFE, which contains 5CQA as the principal component, exhibited satisfactory skin-care properties, including enhanced type I procollagen synthesis and the inhibition of MMP-1 and MMP-3 to MMP-9. The practical manufacturing of CFE loaded in NLCs was successfully achieved in this work using the ANN algorithm coupled with K-fold cross-validation. The optimal formulation offers acceptable qualities of the desired NLCs, including a small particle size, moderate zeta potential value, and high %EE of 5CQA. The experimental values of the target responses were not significantly different compared to the values predicted by the mathematical model. The proportions of CFE, total lipids, liquid lipids, and emulsifiers influenced the physicochemical properties of the NLCs to different degrees. Improved dermal absorption of 5CQA into deeper skin layers with low cytotoxicity and irritation potential is an advantage of NLCs. The fabricated CFE-NLCs remained functionally and chemically stable for up to 90 days of storage at 4°C and 25°C. The proposed ANN models not only offer a potential tool for the production of CFE-NLCs but also are candidates for improving the chemical stability and dermal absorption of CFE-NLCs for further skin-care improvement applications. Prospective studies are needed to address the in vivo anti-skin-aging properties of CFE-NLCs.

## Acknowledgments

 The financial support of the Research Institute of Rangsit University, Thailand (grant number 12, 2018), is gratefully acknowledged.

## Conflict of Interest

 The authors declare no conflicts of interest.

## Ethical Issues

 Not applicable.

## Supplementary Materials


Supplementary file 1 contains Figures S1-S10.
