# Supplementary material for: Artificial Neural Network Modeling of Nanostructured Lipid Carriers Containing 5-O-Caffeoylquinic Acid-Rich Cratoxylum formosum Leaf Extract for Skin Application
Source: Adv Pharm Bull. 2021 Oct 6;12(4):801–17. doi: 10.34172/apb.2022.082 (PMC9675925; doi:10.34172/apb.2022.082)
Supplement: Supplementary file 1 — contains Figures S1-S10. [file apb-12-801-s001.pdf]

Adv Pharm Bull, 2022, 12(4), 818-827
doi: 10.34172/apb.2022.087
https://apb.tbzmed.ac.ir

Preparation and Antiproliferative Activity Evaluation of Juglone-Loaded BSA Nanoparticles

Ali Jahanban-Esfahlan[1], Soodabeh Davaran[2,3], Siavoush Dastmalchi[1,2,4*]

[1]Biotechnology Research Center, Tabriz University of Medical Sciences, Tabriz, Iran.
[2]School of Pharmacy, Tabriz University of Medical Sciences, Tabriz, Iran.
[3]Drug Applied Research Center, Tabriz University of Medical Sciences, Tabriz, Iran.
[4]Faculty of Pharmacy, Near East University, POBOX: 99138, Nicosia, North Cyprus, Mersin 10, Turkey.

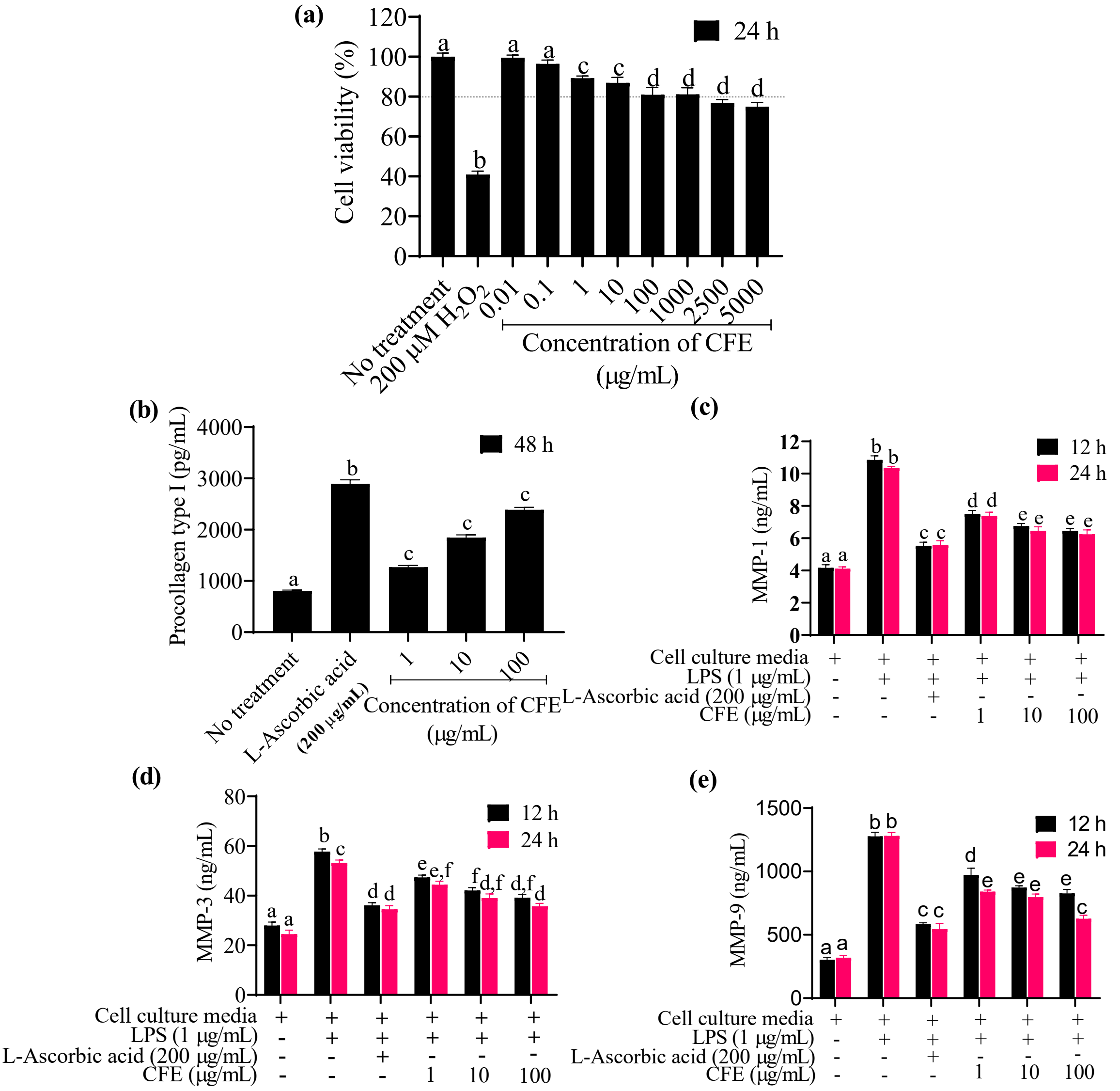

Figure S1. Cytotoxic and biological properties of CFE for cosmetic applications: (**a**) cytotoxicity to HDFn cells, (**b**) procollagen type I synthesis, (**c**) inhibitory activity of CFE against MMP-1, (**d**) inhibitory activity of CFE against MMP-3, and (**e**) inhibitory activity of CFE against MMP-9. Values sharing the same letter above the bars indicate a lack of significant difference ($p > 0.05$).

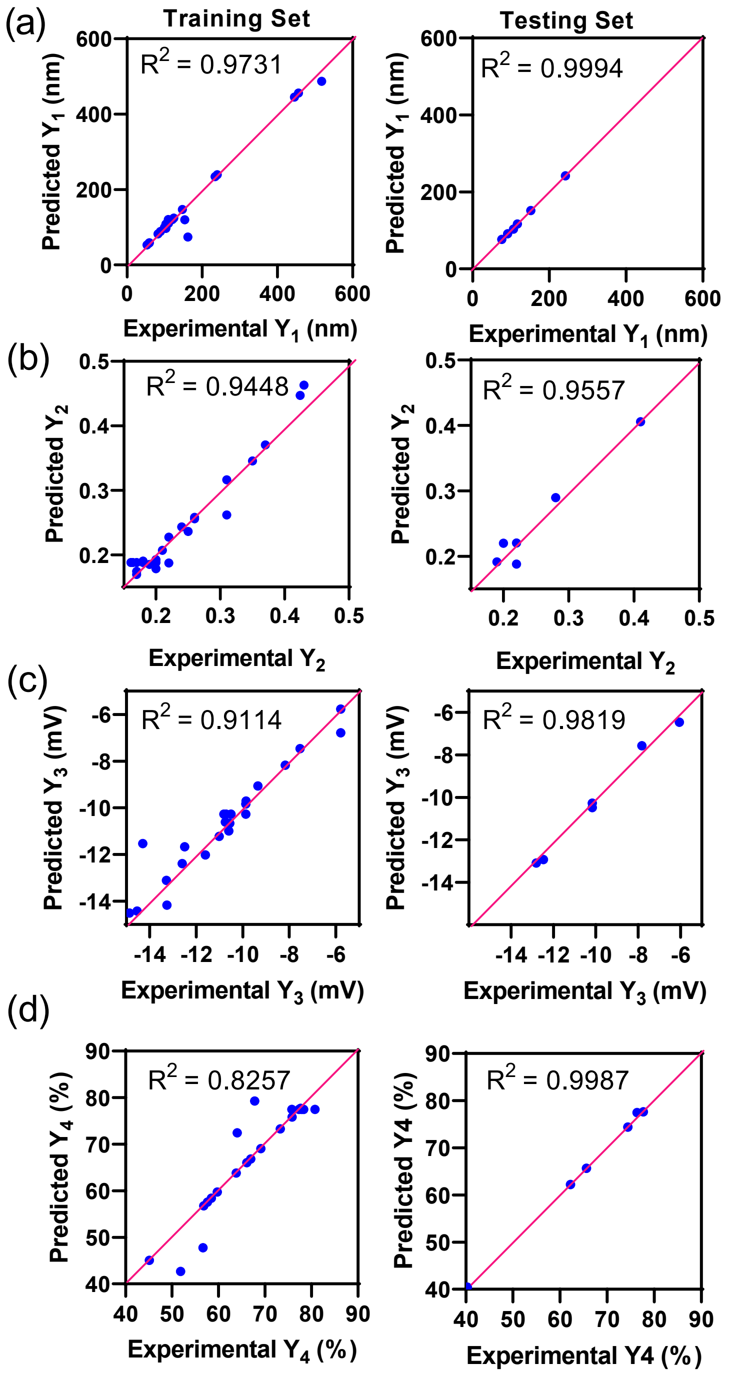

Figure S2. Plots showing the relationship between experimental and predicted values in the training (left panel) and validation sets (right panel): (**a**) hydrodynamic diameter; (**b**) PDI; (**c**) zeta potential; and (**d**) EE% of 5CQA in NLCs.

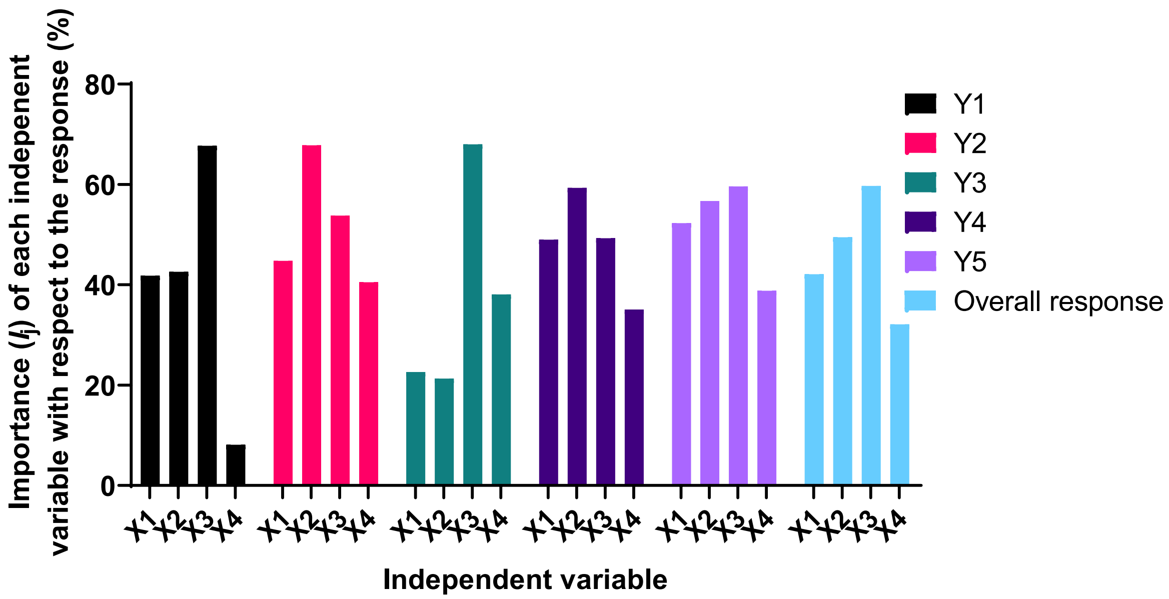

Figure S3. Plots showing the *I*j value of each independent variable with respect to the responses.

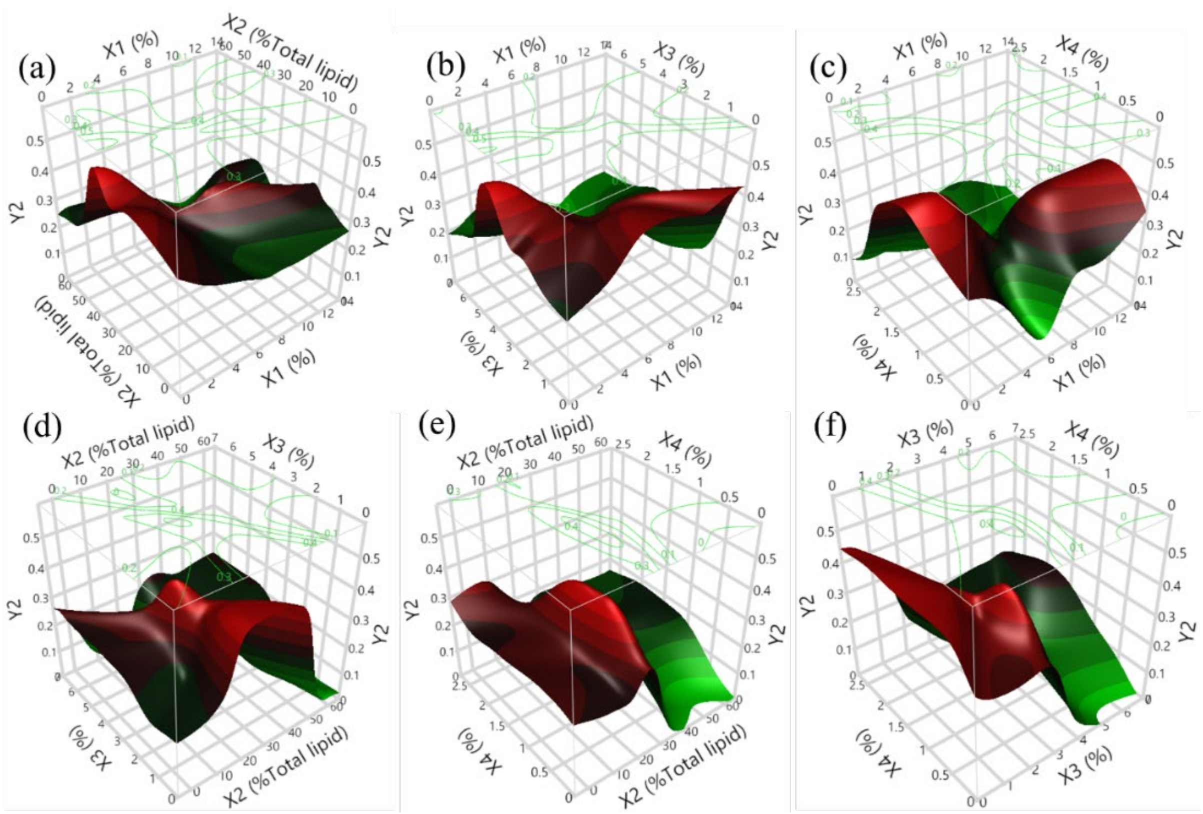

Figure S4. 3D plots showing the combined effects of independent variables on the PDI of CFE-NLCs (refer to Table 1 for X_1–X_4 and Y_2).

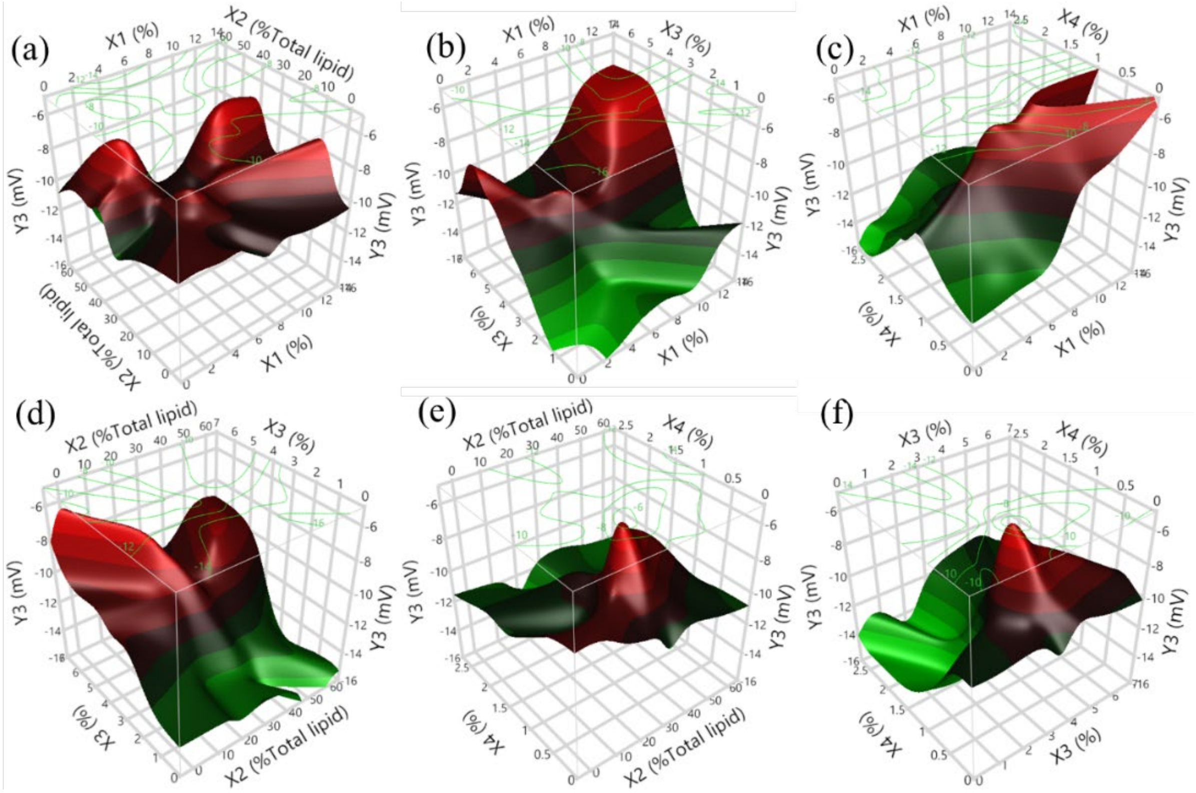

Figure S5. 3D plots showing the combined effects of independent variables on the zeta potential of CFE-NLCs (refer to Table 1 for X_1–X_4 and Y_3).

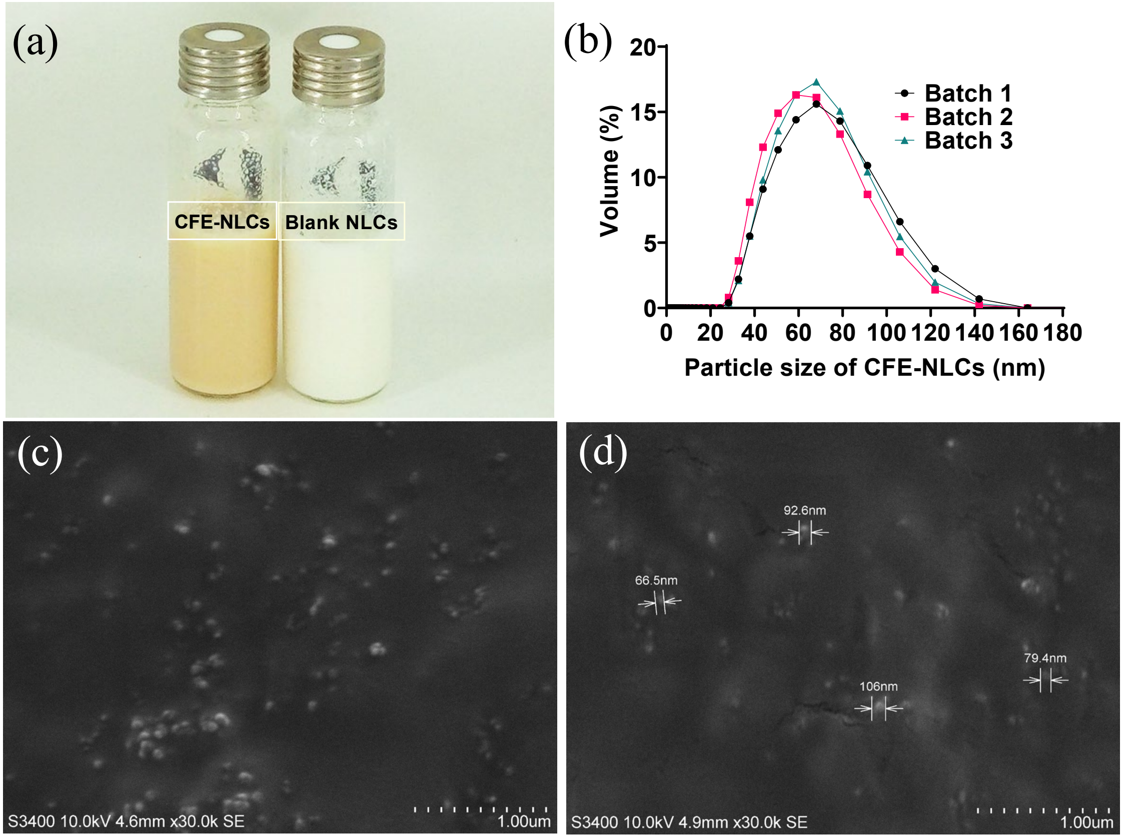

Figure S6. Physical properties of the optimal CFE-NLCs and blank NLC aqueous dispersions prepared under optimized conditions: (**a**) physical appearance; (**b**) hydrodynamic diameter of CFE-NLCs prepared from 3 different batches; (**c**) SEM image of CFE-NLCs; and (**d**) SEM image of blank NLCs.

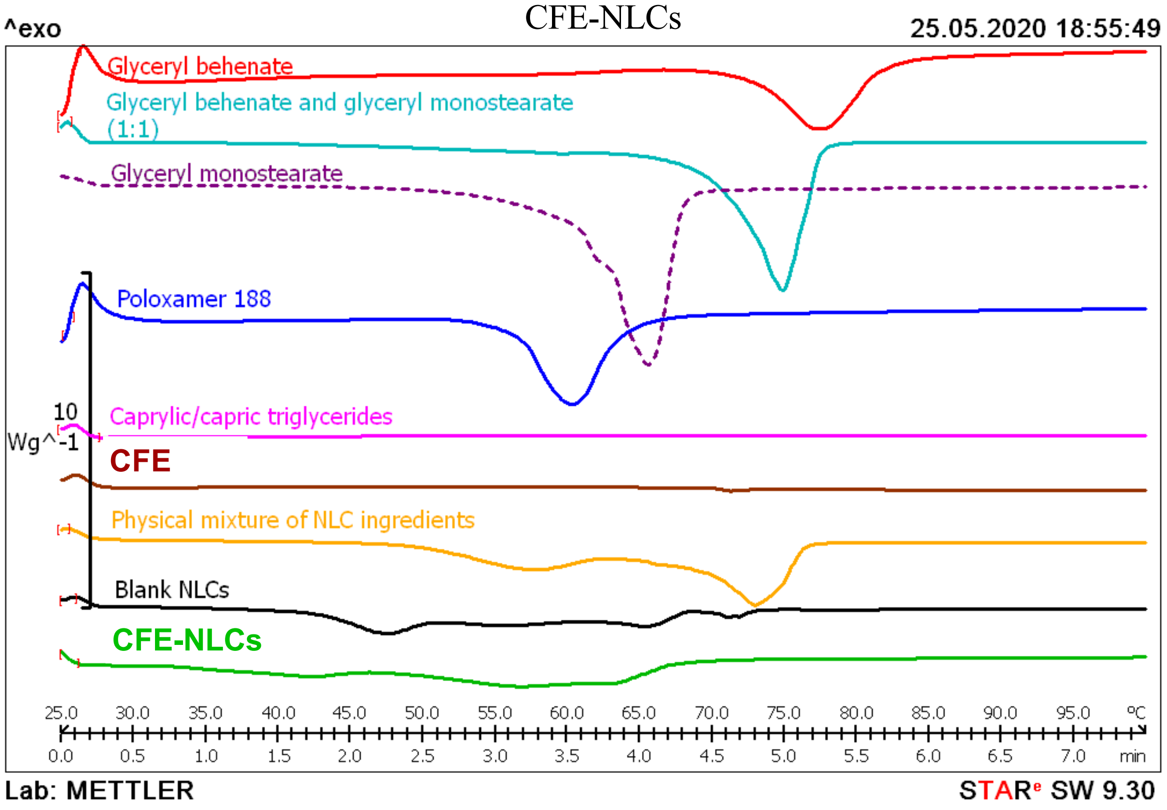

Figure S7. DSC thermograms of CFE-NLCs, blank NLCs and their components.

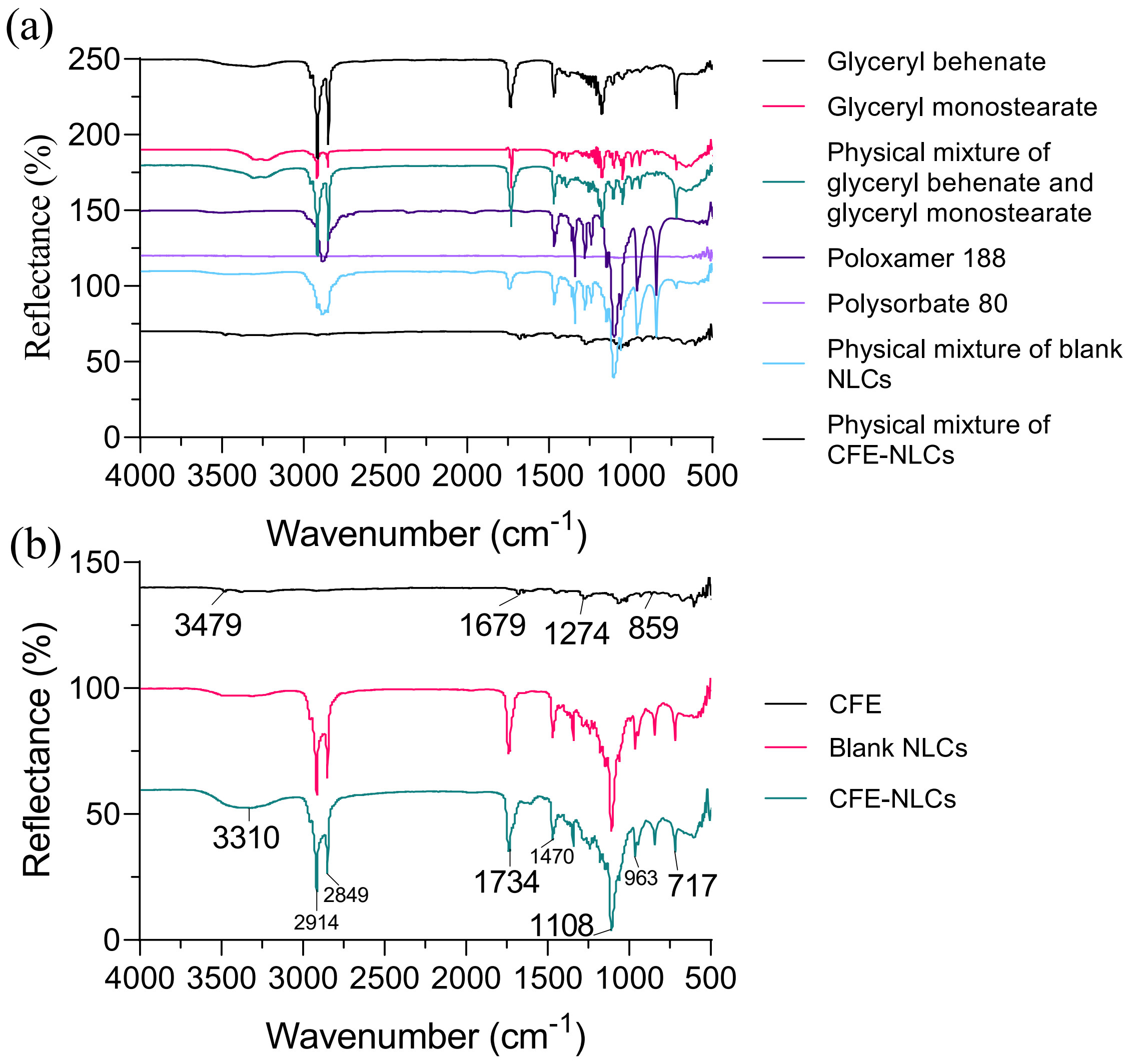

Figure S8. Plots showing ATR-FTIR spectra of (**a**) NLC compositions and (**b**) CFE, CFE-NLCs and blank NLCs.

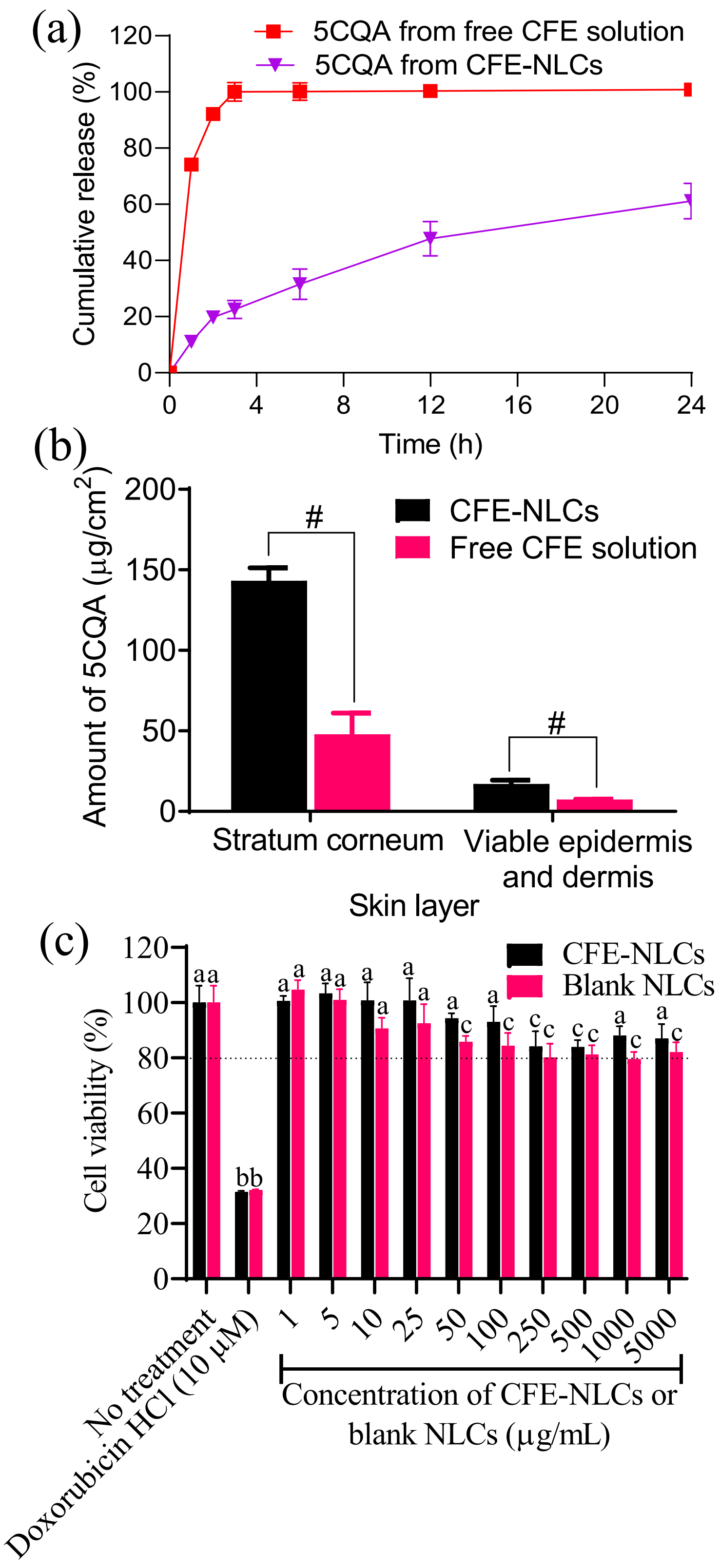

Figure S9. (**a**) In vitro release of total polyphenols and 5CQA. (**b**) In vitro permeation studies showing the amount of 5CQA retained in the stratum corneum and the viable epidermis and epidermis layers after 24 h of incubation ($^{\#}$ $p < 0.001$, $n = 3$). (**c**) Cytotoxicity of CFE-NLCs and of a blank NLC dispersion to HDFn cells ($n = 3$).

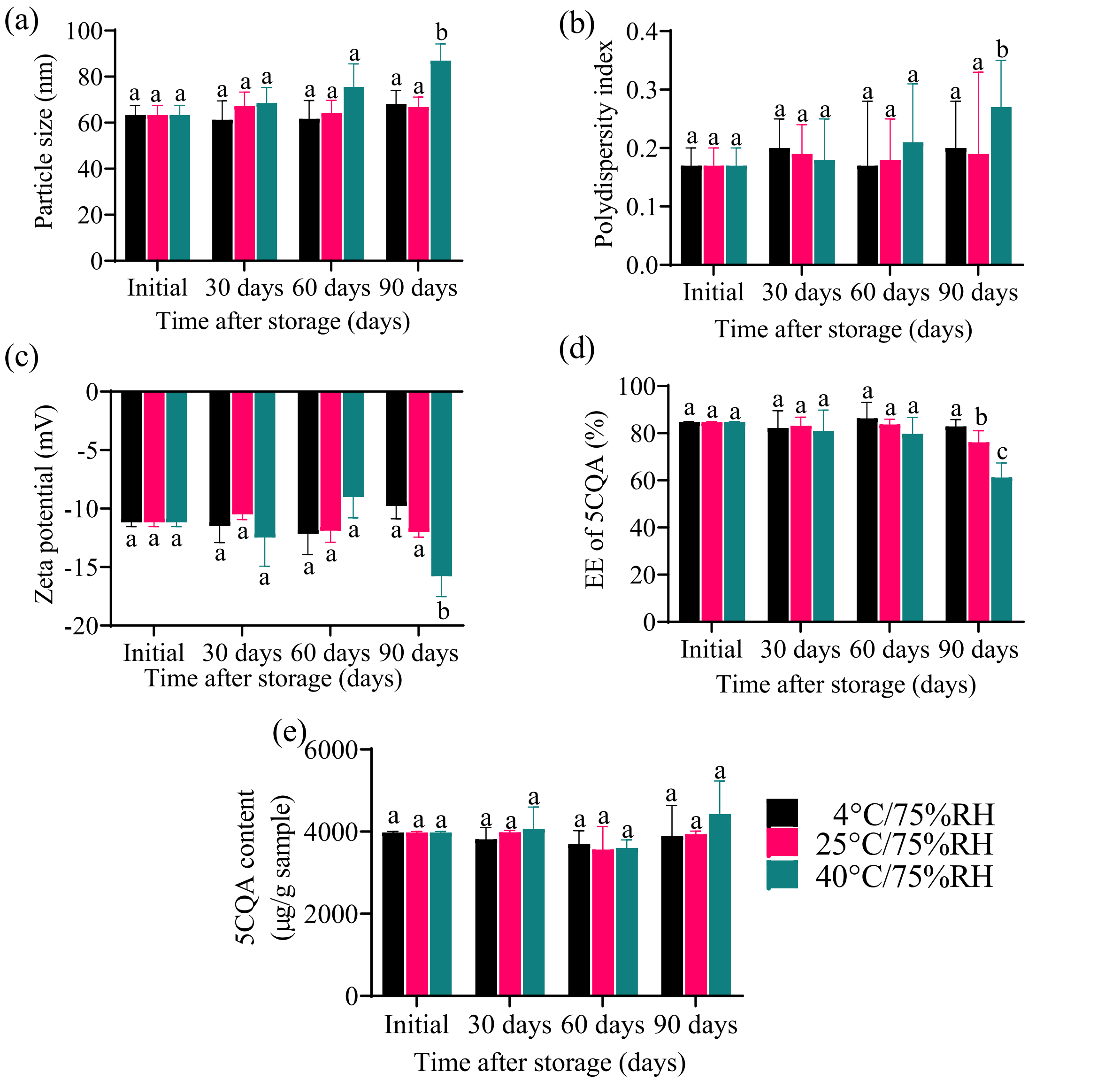

Figure S10. Physicochemical stability of CFE-NLCs and free CFE solution initially and after storage at 4±2°C, 25±2°C and 40±2°C/75±3% relative humidity for 90 days: (**a**) particle size; (**b**) PDI; (**c**) zeta potential; (**d**) %*EE* of 5CQA; and (**e**) content of 5CQA in CFE-NLCs. Values with the same letter above the bar exhibit no significant difference compared with the initial values ($p > 0.05$) ($n = 3$).
